# Auditory perceptual maps in humans and mice share common structures and predict perceptual decisions in discrimination learning

**DOI:** 10.1038/s44271-026-00485-w

**Published:** 2026-06-20

**Authors:** Johannes P.-H. Seiler, Giuseppe Cazzetta, Aida Ghobadi, Simon Rumpel

**Affiliations:** Institute of Physiology, Focus Program Translational Neurosciences, University Medical Center of the Johannes Gutenberg University Mainz, Mainz, Germany

**Keywords:** Perception, Human behaviour, Decision

## Abstract

To efficiently perceive sensory information and guide behavior, the brain organizes incoming sensory stimuli into internal maps that capture perceptual relatedness between stimuli. Whether these maps, typically assessed in scaling paradigms without feedback, also shape perceptual decisions during reinforcement-based conditions remains unclear. Here, we assess task-naïve perceptual maps from similarity judgments of pulsed sound stimuli, and compare them to perceptual maps obtained from multiple discrimination tasks in humans (n = 152) and mice (n = 11). We find that task-naïve maps predict how well humans discriminate sounds, how quickly they learn, and which stimulus features guide their choices. Moreover, naïve and task-based maps share key structures, suggesting a stable perceptual map architecture throughout learning. Remarkably, human task-based and task-naïve perceptual maps share key features and structures with the task-based perceptual maps of mice, indicating congruent structures of auditory perception across species. Together, our results indicate that perception relies on robust internal maps that provide a common framework to flexibly guide behavior in changing environments.

## Abstract (web summary)

How individual perceptual maps shape behavior remains unclear. The current study compares task-naïve and task-based auditory maps in humans and mice, finding congruent perceptual structures across conditions and species, guiding perceptual learning.

## Introduction

In order to efficiently navigate natural environments, individuals are required to continuously process incoming information from the outside world and react with adequate behavioral decisions. The brain represents this incoming information internally, arranging various inputs according to their level of relatedness resulting in a representation in the form of a map^1,2^. Such *representational maps*^3–6^, not only allow to discern different sensory entities, but also encode the relations between them. For instance, at a perceptual level in which phonetic relatedness is encoded, the words ‘light’ and ‘night’ would be expected to be mapped closely together, whereas at a map encoding semantic relatedness, these two words would be mapped more distinctly. Thus, representational maps can encode multiple different stimulus features, effectively capturing the complex information of multidimensional stimuli to guide subsequent behavior^7–9^. Besides their implications on individual behavior, representational maps serve as an instrumental approach to compare encoded sensory information between different species. For instance, a previous study comparing the representational map structures in humans and monkeys identified shared visual representations of animate or inanimate objects^10^. Similar cross-species comparison of representational maps appears promising for the auditory system, in which a high degree of functional convergence and gene homology has been described^11–13^.

Originally developed in the cognitive sciences, the abstract concept of representational maps describes the organization and encoding of the relatedness between representational tokens in cognitive space at various levels of hierarchy. In experiments, the structure of representational maps has been classically assessed in the psychophysical domain, e.g. based on simple ratings of perceived similarity^14–16^. Over recent years this approach has also found broad support in the neurosciences^4,17^. Specifically, it was shown that perceptual representational maps assessed by self-reports or behavioral similarity estimates, share key structures with neuronal representational maps^8,18–20^, assessed by comparing the similarities between stimulus-related patterns of brain activity^10^. In this manuscript, we use the term *perceptual map* to refer to the representational map that encodes perceptual relatedness of the sound stimuli used in this study, experimentally estimated by assessing their perceived similarities. Specifically, these perceptual maps are estimated from individual representational similarity matrices, comprising all individual pairwise similarity values between a defined set of stimuli, adhering to the procedures of previous studies (see ref.^4^ for a review).

Typically, assessments of perceptual maps are conducted under basal conditions, without the context of a task and without reinforcement. In contrast, studies on perceptual decision-making across species often employ tasks in which reinforcement is crucial^21^. Thus, it remains unclear in how far perceptual maps estimated in different contexts are related. Do naïve and task-based perception diverge, suggesting the implementation of independent perceptual maps for both conditions? Or to what extent is it possible to predict task-specific behaviors from naïve perceptual maps, suggesting a common map being employed across conditions? In addition, are principles of how perceptual maps determine choice behavior shared across mammals?

In this study, we address these questions, using the auditory system as a model to investigate the perception of pulsed sound stimuli in humans and mice. We conduct assessments of task-naïve perceptual maps using a reinforcement-free scaling paradigm in humans, asking participants to rate the perceived pairwise similarities for a fixed set of sounds. In addition, we independently assess task-based perceptual maps, using reinforcement learning to categorically discriminate sound stimuli. In these reinforcement-based discrimination tasks participants learn to classify sounds into two categories defined by a specific sound property in order to obtain monetary reward. We parametrize the sounds by a defined set of stimulus features, and test to what degree these features are represented in naïve and task-based perceptual maps. Reinforcing different sound features in the discrimination task, we measure how individuals adapt their behavior and test if this adaptation is predicted by naïve perceptual properties. Observing a general conservation of map structures in naïve and task-based conditions in humans, we conduct an equivalent discrimination task in mice, observing a high level of conservation across species. Lastly, we test the adaptation of decision strategies over the course of learning in humans and mice, finding that task performance is determined by an increase in the impact of reinforced stimulus features while stimulus-independent choice factors become suppressed.

## Methods

### Pulsed sound stimuli

For our human study, we used a set of 20 pulsed sound stimuli (Supplementary Fig. 1a, see ref. ^22^). Each of those sounds consisted of a 1s-long interval with a random temporal pattern of short white noise pulses (single pulse duration: 30 ms with a linear up- and down-ramp of 5 ms; the temporal pattern of pulses was randomized with a constrained minimal interval of 50 ms between consecutive pulses). We systematically created pulsed sounds with varying numbers of pulses, ranging over nine categories from 3/s up to 11/s. The sounds for our human experiment were selected to cover all available numbers of pulses per second, and to vary in their temporal pulse patterns. Furthermore, the sounds were selected based on comparable distinctions along three different stimulus features – *pulses per second*, *irregularity of accents* and *pulse imbalance* – which were used to characterize the stimuli (see Methods below, Supplementary Fig. 1b-d). For our mouse study, we used a larger stimulus set of 90 pulsed sound stimuli, incorporating the 20 stimuli used in our human study (Supplementary Fig. 3a, see ref. ^23^). For each pulse category, this larger stimulus set comprised 10 sounds with different random temporal patterns.

### Sound features of pulsed sound stimuli

We characterized all pulsed sounds according to a set of three interpretable and diverse features, which were determined by systematic literature research. In a previous study, we used in vivo two-photon calcium imaging of population activity patterns in the auditory cortex of passively listening mice in response to the same set of pulsed sound stimuli, and found that the representational structure at the neuronal level captures entangled properties of the pulsed sounds described by the three stimulus features reflecting major aspects of the perceptual map^23^.

1. *Pulses per second*: Overall sum of white noise pulses in the respective pulsed sound^24,25^. This number ranged from 3 up to 11 and was equivalent to the pulse category of the sound. The number of pulses per second provides a metric of the aggregate acoustic energy of a pulsed sound.
2. *Irregularity of accents*: We counted all temporally defined clusters of pulses in each respective sound, providing a proxy of groups of rhythmic accents^26,27^. For the grouping, we used a threshold interval of 50 ms (adapted from psychophysical studies^28^) between consecutive pulses, where all pulses falling below this threshold were grouped together into one *accent*. Then, we quantified the irregularity of accents by computing the standard deviation of the time intervals between consecutive accents in each sound.
3. *Pulse imbalance*: The ratio of the number of pulses in the first half of the stimulus and the number of pulses in the second half of the stimulus, providing a measure of the temporal dynamics of stimulus energy^29^.

We correlated the feature values of our full set of sounds (mouse study) and the subset of sounds (human study), respectively, observing only weak associations between the different sound features.

### Probing human perception of pulsed sounds in an auditory two-alternative forced choice task

#### Human sample

A total number of 162 healthy students from the University of Mainz was recruited to participate in our study, using an online recruiting system^30^. Exclusion criteria were the presence of impaired hearing ability, active psychiatric or neurological disorders, or insufficient German language skills, all assessed through self-reports (see general information questionnaire below). From the initial sample, 10 participants encountered technical problems during the experiment, leading to incomplete data and exclusion from the analyses presented here. Thus, a final sample of 152 participants was used for the analyses of this study. The sample comprised 120 women (78.9%) and 32 men (21.1%), with an average age of 22.8 years (for further demographic information see Supplementary Table 1). Gender and age information was assessed through self-reports.

#### General procedure

The human study was approved by the local ethics committee (Ethikkommission der Landesärztekammer Rheinland-Pfalz, processing number 2024–17477). There was no pre-registration of the study.

The experiment of the study was conducted on a single day in the facilities of the Mainz Behavioral and Experimental Laboratory (MABELLA) as part of a larger study on perception and associative thinking. Written informed consent was obtained from all participants of the study. After receiving an introduction to the study and providing consent, participants started the experiment, implemented in a custom MATLAB^®^ program which was presented on a standard computer screen. For the duration of the experiment, participants were instructed to wear headphones, which were also used to deliver the auditory stimuli. The study comprised different steps listed in the following. Experimental assessments from this study unrelated to the perception of pulsed sounds, are analyzed in previous publications^22,31^ and are listed at the end of this section.

#### Psychometric questionnaires

First, participants were asked to fill out a battery of different self-report scales in order to assess demographic and psychometric properties, such as personality traits and properties of mental health and resilience, to ensure that they matched the inclusion criteria. Specifically, participants reported *general information* (gender, age, weight, size, patient history), Big Five personality characteristics (*BFI-10*: Big Five Inventory^32^ covering neuroticism, extraversion, openness, agreeableness, conscientiousness), trait anxiety (*STAI-Y*: State Trait Anxiety Inventory^33^), indicators of mental resilience (*BRS*: Brief Resilience Scale^34,35^), boredom proneness (*BPS*: Boredom Proneness Scale^36,37^) and state boredom at the start of the experiment (*MSBS*: Multidimensional State Boredom Scale^37,38^). All questionnaires were presented in German language.

#### Auditory similarity ratings to assess task-naïve perceptual maps

Next, we presented participants with a scaling paradigm to rate the subjectively perceived similarities between 20 pulsed sounds in a task-naïve setting free from reinforcement. The scaling paradigm started with an initial presentation of all sounds of the stimulus set (twice repeating the 20 pulsed sounds in a random order) to familiarize participants with the stimulus set and its variety. During this habituation, the interval for stimulus presentation was set to 1.2 s with an inter-trial interval of 1.5 s. In a next step, random pairs of stimuli were consecutively presented to the participants with an interval of 1.5 s in between. After the presentation of each pair of sounds, a slider was displayed on the screen with the request to rate the similarity between both presented stimuli on a visual analog scale (VAS) from “not similar at all” to “completely identical” (Prompt: “Please rate the similarity between the two sampled sound stimuli.”). Participants had no time limit for moving the slider and rating the similarity. After moving the slider, participants were able to submit their response, which led to the start of the next trial, presenting another pair of sounds after an inter-trial interval of 1.5 s. For each trial, the slider was initialized with an intermediate neutral similarity value and had to be actively moved in order to enable the submission of the response. The 20 pulsed sounds presented in the task resulted in 210 trials for each participant to rate all pairs of stimuli, including pairs of identical stimuli. The order of all pairs of stimuli and the order of presenting the stimuli within each trial were randomized.

#### Auditory two-alternative forced-choice task to assess task-based perceptual maps

In a next step, participants underwent three independent rounds of a two-alternative forced choice task, in which participants needed to discriminate pulsed sound stimuli according to different rules in order to obtain reinforcement that translated into monetary reward at the end of the experiment. In each trial of the task, participants were first presented with a pulsed sound stimulus, during which the neutral fixation cross (white color) on the screen disappeared for one second. The sound presentation was followed by a response window of up to 2 s, in which participants had to respond to the stimulus by one of two possible key presses (left and right arrow key, marked on the keyboard of each participant). This response window was also cued by a re-appearance and a color change of the fixation cross to yellow. After making a decision and pressing a key, subjects received short visual feedback about their response to enable learning (0.5s-long color change of the fixation cross to blue in the case of a correct choice, or to red in the case of a false choice). After an inter-trial interval of 2 s, the next trial was initiated, starting this cycle of steps again. Participants were instructed about these principles in detail before beginning the task (main task prompt: “You will be presented with different sound stimuli that need to be responded with a press on one of the two marked keys. After each response, you will receive short feedback if your response was correct or not. It is on you to identify the correct classification rule of each task.”). Moreover, subjects were told that the number of correct choices in the task determined a part of their monetary expense allowance at the end of the experiment (€ 10 fixed plus € 1-15 based on task performance) in order to incentivize them to adequately engage in the task, and that each round of the task had an independent rule. Each round of the task ended after completing 170 trials. The discrimination rules in each round of the task followed a separation of the sounds by the three different sound features *pulses per second*, *irregularity of accents* and *pulse imbalance* (see Supplementary Fig. 1b–d). Participants were explicitly instructed that all three discrimination tasks were independent from each other. Specifically, for each sound feature, the nine pulsed sound stimuli which scored low on that feature were assigned to one choice alternative in the task, whereas the nine pulsed sounds scoring high on that feature were assigned to the other choice alternative. The two pulsed sounds with intermediate feature scores were randomly rewarded. While the separation of pulsed sound stimuli according to their features was equivalent for all subjects, the particular assignment to the choice alternatives (i.e. to the right or left arrow key) was randomized to balance out potential choice biases. The temporal order of the three different task conditions (i.e. the three features determining the task rules) was also randomized for each participant. In each condition, the sequence of presented sounds was random, however with a constraint that at the beginning of each task, only the eight sounds with either very high or very low scores on the respective feature were presented in order to enhance learning under conditions of good discriminability. Over the progression of the task, subjects were increasingly presented with more ambiguous pulsed sounds. After the end of each discrimination task, participants were asked to describe the reinforcement rule of the completed task in their own words.

#### Additional elements of the experiment, not considered in this study

The assessment of auditory perception of pulsed sound stimuli in our study was integrated into a larger experiment that each participant underwent. In addition to the abovementioned experimental steps, participants also completed a battery language-based divergent thinking tasks^39,40^ as well as a semantic scaling paradigm, rating the pairwise similarities of set of word stimuli^22^. After completing the semantic similarity ratings, participants also filled out some questions about their perception and sentiment of five short texts^31^. The other steps of the experiment are conceptually widely independent from our analyses presented here. Thus, the data of the above subparts is not considered for the present study, but was used for independent studies on auditory perception and creativity^22^ as well as on the perception of information from semantic stimuli and boredom^31^. In this context, the naïve perceptual similarity matrices obtained from the scaling paradigm with pulsed sound stimuli have been previously published, however in the context of a different and independent research question concerning their relation to verbal creativity assessments^22^. Likewise, the psychometric questionnaire data was previously published in order to address other research questions that do not overlap with the analyses presented here^22,31^.

### Probing mouse perception of pulsed sounds in an auditory-cued go/no-go task

To compare the discrimination of pulsed sound stimuli in humans with equivalent discrimination in mice, we used a behavioral dataset from our laboratory in which mice had to discriminate a large set of pulsed sound stimuli based on their number of pulses (see Methods above). Parts of this dataset was used  for a separate study in which we analyzed sound-specific decision biases and related these biases to neuronal representations of pulsed sound stimuli in independent mice^23^, however all analyses here are independent from the other study. In this study, we focus on the perceptual discrimination of the subset of sounds shared between the mouse and human tasks, and on a cross-species comparison of average perceptual decisions.

All animal experiments were performed in accordance with the German laboratory animal law guidelines for animal research and had been approved by the Landesuntersuchungsamt Rheinland-Pfalz (Approval # 23 177-07/G 17-1-051).

#### Mouse sample

We obtained an initial sample of 12 wildtype C57BL/6 J mice with an age of approximately 6 weeks. Before the discrimination task, mice were habituated to the experimenter for one week. Mice were kept in groups of four, and housed in 530 cm^2^ cages on a 12 h light/dark cycle with unlimited access to dry food. Experiments were carried out during the light period.

#### Behavioral setup

For the go/no-go discrimination task, we used four identical Skinner boxes (H10-11M-TC, modified by Coulbourn Instruments, Whitehall, PA, USA) with a soundproof isolation cubicle. The boxes had an ambient illumination provided by a warm-white LED. For Sound delivery, we used an ASUS Xonar DX, PCI 7.1 Audio card, with an external amplifier (SLA-1, by Applied Research and Technology, TEAC Europe GmbH, TASCAM Division Wiesbaden, Germany), a modified equalizer (Applied Research and Technology, TEAC Europe GmbH, TASCAM Division Wiesbaden, Germany) and a custom-made mono loudspeaker. Each Skinner box contained a port with two holes (one for water delivery, one for air puff emission). The water delivery was controlled by an electric valve, connected to a water dispenser (H20-94, by Coulbourn Instruments, Whitehall, PA, USA) via a small silicone tube. Poking responses to the port were measured using an infrared beam sensor (H24-01R, Coulbourn Instruments, Whitehall, PA, USA), which allowed us to register the poking behavior of mice approaching the water dispenser. Air puff emission was controlled by another electric valve and comprised a pressure of 4 Bars. Above the water dispenser, a blue LED was mounted, serving as additional cue.

#### Auditory go/no-go task to assess task-based perceptual maps in mice

To test the auditory perception of mice in the form of the discrimination of pulsed sound stimuli, we conducted an auditory-cued go/no-go task following an established protocol^23,41,42^. Here, mice were water-deprived and then admitted to the go/no-go task, where they learned to differentially react to different pulsed sounds in order to obtain water reward (so called “go trials” indicated by “go stimuli” S + ) and avoid an aversive air puff (so called “no-go trials” indicated by “no-go stimuli” S-). The water deprivation followed established standard protocols motivating subjects to engage in the task without causing notable behavioral impairments^23,41,42^. Animal welfare during the deprivation periods was ensured through regular visual inspections and weight controls. In the task, mice iteratively underwent multiple trials that followed a general structure: (1) By poking into the licking port, mice self-initiated the start of a trial and the emission of a sound (S+ or S-) via the loudspeaker (sound interval: 1 s). (2) This interval was followed by a response window of 1 s in which we quantified the fraction of time for which the mouse was poking into the port. If the fraction of poking exceeded a threshold of 70%, the trial was counted as a valid poke (“go response”), whereas a fraction of poking <70% was counted as no poke (“no-go response”). (3) Depending on the trial type and poking response the mouse received feedback for its behavior. In case of a go trial and a go response (“hit”), the water valve was opened for 20 ms, emitting a small water reward ($\sim$5 μL). In case of a go trial and a no-go response (“miss”), no reward was emitted without any further consequence. In case of a no-go trial and a go response (“false alarm”) the air valve was opened emitting an air puff as punishment and a time-out of 6 s. In case of a no-go trial and a no-go response (“correct rejection”), mice did not receive any further consequence. (4) Next, an inactive inter-trial interval started (2–4 s, randomly selected from a uniform distribution) in which the mice had to withdraw from the licking port. After this cycle, mice were able to start a new trial (inducing the start of step 1). During steps 1–3, the blue LED was turned on, serving as a general cue of trial onset. The pulsed sounds were assigned to the different contingencies such that the number of *pulses per second* served as key feature to signal their meaning: Sounds with 3-6 pulses per second served as S- (no-go stimuli) indicating that mice should retract from the licking port, whereas sounds with 8–11 pulses per second served as S+ (go stimuli) indicating the availability of a water reward upon poking. Stimuli with 7 pulses per second were neither reinforced, nor punished (“off-target”). For the discrimination task, we used a stimulus set of 90 pulsed sounds (10 sounds from each respective category of 3–11 pulses/s, Supplementary Fig. 3a). The order of S+ and S- was randomized with the constraint of an equal number of go and no-go trials every eight trials. Stimulus presentations within the groups of S+ and S- were also randomized. Mice were admitted to two task sessions per day, where each session lasted 30 min, corresponding to approximately 150–250 trials.

#### Task procedure

After a water-deprivation of 24 h, mice underwent a shaping phase of two weeks, in which we only exposed them to S+ stimuli from the 11 *pulses per second* category. In this phase, mice initially received a water reward easily, even if their poke was only short. Then, over sessions we gradually increased the threshold for poking (up to a final threshold of 70%, see above) to train animals to a reliable poking response. In the next phase, mice were presented with the full task, comprising S+ and S- sounds in equal fractions. However, in order to facilitate initial learning, only sounds from the categories of 3 *pulses per second* or 11 *pulses per second* were presented. After a mouse reached a performance threshold of 70% correct responses for at least 2 sessions, mice transitioned to the next phase, where we expanded the set of presented sound stimuli to also cover 4, 5, 9, 10 *pulses per second*. Typically, the initial learning took mice around 1000–2000 trials corresponding to approximately 1-2 weeks. In this second task phase, if mice again reached a performance threshold of 70% correct trials for two consecutive sessions, they transitioned to the final phase including the full set of pulsed sounds from 3 to 11 *pulses per second*. Go and no-go trials were presented in a pseudorandom order, ensuring no more than four consecutive repeats of the same trial type. Due to a technical error in the randomization process, some of the 90 pulsed sounds used in the study were only presented on very few trials and therefore excluded from our analysis. This led to an overlapping dataset for 19 of the 20 sounds used in both the human and mouse experiment. From our sample, one mouse did not learn the initial discrimination and hence was excluded, leading to a total of *n* = 11 mice considered in our analysis.

### Statistical analysis

All analyses were conducted using the MATLAB^®^ statistics and machine learning toolbox (The Mathworks Inc., Natick, Massachusetts, USA, version R2022a). All data was pseudonymized before analyzing. All hypothesis tests were performed in a two-sided manner, if not specified differently. Reported confidence intervals (CI) refer to 95%-range.

#### Psychometric questionnaires

The self-reported data was analyzed by computing the sum score for each questionnaire. Participants that accidentally skipped single items of a questionnaire or subscale were excluded from the respective analysis.

#### Human perceptual discrimination in the two-alternative forced choice task

To assess discrimination performance of humans in the two-alternative forced choice task, we used the data of all trials in each task condition. This procedure resulted in a binary vector for each mouse with its length reflecting the number of trials in the learning process, where each element represents the decision outcome of a given trial (false/correct). To estimate the performance over trials, we computed the moving mean over the vector of binary decision outcomes for each participant, with a moving bin size of 8 trials. To compute the response probability for each sound, we considered all trials in each respective condition and computed the choices to a reference alternative across individuals and task features, namely to the choice alternative that was associated with higher values in the respective sound feature. This expression of response probability relative to a reference alternative made it possible to compare decisions across subjects and task conditions. Based on the response probability to a reference alternative (${p}_{{resp}}$), we estimated the perceptual similarity between two sounds in the discrimination task as $1-\Delta {p}_{{resp}}$.

#### Analysis of learning dynamics in humans

To quantify the individual human learning behavior in each of the task conditions, we used the individual performance vector over all trials $t$ and fitted it with a sigmoid function: ${performance}\left(t\right)=\frac{m-0.5-b}{1+{10}^{-k* \left(x-{x}_{0}\right)}}+0.5+b$. In this equation, $m$ reflects the maximal performance, $b$ reflects an initial bias expressing an offset from chance performance, $k$ reflects the skewness of the learning curve and ${x}_{0}$ reflects the shift of the sigmoid to the right, i.e. the number of trials for which no learning is observable. We then fitted the sigmoid to the data of each participant using a non-linear least squares algorithm. We constrained the fit to plausible values of all parameters, leading to reliable fits (*m*: [0.5 1], *b*: [-0.5 0], *k*: [0 0.1], *x*_*0*_: [0 200]). We verified that these sigmoid fits of learning curves explained our data better than an alternative simple linear model by assessing the fraction of explained variance (*n* = 152 participants; mean R^2^ ± SD: pulses, sigmoid 0.118 ± 0.254 vs. linear 0.104 ± 0.115, irregularity, sigmoid 0.140 ± 0.174 vs. linear 0.090 ± 0.105, imbalance, sigmoid 0.101 ± 0.157 vs. linear 0.068 ± 0.100), as well as Akaike’s Information Criterion (mean AIC ± SD: pulses, sigmoid −750 ± 80 vs. linear −743 ± 95, irregularity, sigmoid −620 ± 56 vs. linear −611 ± 50, imbalance, sigmoid −608 ± 66 vs. linear −601 ± 40). Based on the fitted learning curve, we then computed the number of trials that it took an individual until reaching a performance of 70% correct (*trials of learning*), as well as the number of trials for the individual learning curve to reach from 20% of maximal performance to 80% of maximal performance (*ramp duration*). For the parameter estimation, we extrapolated the empirically observed learning curves. Together with the fitted $m$ values, these scores effectively capture the maximal learning success ($m$) as well as the steepness of the learning curve (*ramp duration)* and the overall time to learn (*trials of learning*) of individuals, and were compared between task conditions using Wilcoxon rank sum tests.

#### Fitting a psychometric curve to human perceptual decisions

To quantify the individual human perception of pulsed sound stimuli in each of the task conditions, we used the individual response probabilities over all experienced sound stimuli to fit a psychometric curve^43^, expressing choice probability as a function of stimulus properties. Specifically, for each sound feature, we normalized the feature values across all sounds to the range of feature values in our stimulus set. This operation provided normalized metrics of stimulus evidence for all of the sounds, where an evidence value of 0 would indicate that a sound is characterized by the lowest possible value in the current feature, an evidence value of 1 would indicate that a sound is characterized by the highest possible value in the current feature, and a value of 0.5 would indicate that a sound is highly ambiguous and cannot be clearly interpreted in respect to the current feature (lowest and highest possible values refer to the used stimulus set). In particular, we fitted the individual psychometric curves with a sigmoid function: ${performance}\left(t\right)=\frac{m-b}{1+{10}^{-k* \left(x-{x}_{0}\right)}}+b$. In this equation, $m$ reflects the maximal response probability, $b$ reflects a bias of the minimal choice probability from zero, $k$ reflects the skewness of the psychometric curve and ${x}_{0}$ reflects the shift of the curve to the right, expressing a tendency to prefer one alternative over the other for ambiguous stimuli. We fitted the sigmoid to the data of each participant using a non-linear least squares algorithm. We constrained the fit to plausible parameter ranges, leading to reliable fits (*m*: [0.5 1], *b*: [0 0.5], *k*: [0 10], *x*_*0*_: [0.25 0.75]). Based on the fitted psychometric curves, we then compared the psychometric *range* of each individual (calculated as $m-b$) and the *k* and *x*_*0*_ values across task conditions. These metrics allow to compare the maximal discriminability of sounds along a given feature (*range*), the capability to form distinct perceptual decisions based on the given sensory evidence (*k*), as well as choice biases to systematically over- or underestimate the sensory evidence of a given sound feature (x_0_).

#### Explicit understanding of the rules in the discrimination tasks

After each round of the discrimination task, participants submitted their individual estimation of the rule in the previously completed task. From these responses, we manually evaluated whether participants explicitly understood the task rules. For instance, responses such as “Many clicks -> right. Few clicks -> left” were counted as correct for the task based on *pulses per second*. On the other hand, a response such as “The decision rule was about the regularity of the sounds” was counted as correct for the task based on *irregularity of accents*. For the task based on *pulse imbalance* responses such as “Whenever the most of the sounds appeared in the beginning, I had to press left, elsewise, I had to press right” were counted as correct. The evaluation of the free text responses was performed in a blinded fashion by an expert of the study, and was repeated two times to ensure reliability of the evaluation.

#### Perceptual discrimination of mice in the auditory go/no-go task

To assess discrimination performance of mice in the go/no-go task, we concatenated the data of all trials from all completed task sessions for each mouse, where the first and last 5 trials of each session were omitted. In analogy to our human analysis, this procedure provided a binary vector for each individual with its length reflecting the number of trials, where each element represents the decision outcome of a given trial (false/correct). To estimate the performance over trials, we computed the moving mean over the vector of binary decision outcomes for each mouse, with a moving bin size of 100 trials. To compute the response probability for each sound, we considered all trials after the animals had passed the initial learning threshold of ≥70% correct for at least two consecutive sessions, and computed the mean poking probability for all trials in which a given sound was presented. The difference between the mean poking probabilities $\Delta {p}_{{poke}}$ for two sounds provided a general estimate of their perceptual dissimilarity. Conversely, we computed the behavioral similarity of two stimuli in the discrimination task as $1-\Delta {p}_{{poke}}$.

#### Construction of similarity matrices from similarity ratings and discrimination task data

Based on the individual similarity ratings of pairs of pulsed sounds on a visual analog scale in the scaling paradigm, we constructed a similarity matrix, expressing the pairwise similarities of all pulsed sounds in our human stimulus set (20 different pulsed sounds, see Methods above). Following this approach, we constructed structurally comparable similarity matrices from the behavioral similarity estimates in the discrimination task ($1-\Delta {p}_{{resp}}$) for each subject and task condition, respectively. Specifically, we computed the mean response probabilities for each sound in the task. For the human data, we used the data of each choice task to build the mean over all trials in which a given stimulus was presented. For the mouse data, we did the same, however using only the data after the mice had learned the initial discrimination, showing an average performance ≥70% for two consecutive sessions. For the construction of similarity matrices, we assumed that two sounds that are perceived as similar should also show similar behavioral response probabilities. In analogy to this approach, we constructed equivalent similarity matrices for our mouse sample, by considering the poking probabilities to the 19 pulsed sounds shared between experiments and presented to humans and mice in a sufficient number of trials. To obtain general similarity estimates across subjects, we then built the average similarity matrix over all subjects for each of the following conditions: human similarity ratings, human discrimination task with *pulses per second*, human discrimination task with *irregularity of accents*, human discrimination task with *pulse imbalance*, mouse discrimination task with *pulses per second*.

#### Clustering similarity matrices obtained from the similarity ratings

To determine the number of clusters that best described the perceptual similarity ratings in the scaling paradigm, we performed k-means clustering algorithm with an evaluation using the Calinski-Harabasz criterion (CH). The evaluation was performed for a range of cluster solutions from k = 1 to k = 10. The Calinski-Harabasz index, which measures the ratio of between-cluster variance to within-cluster variance, was used to assess cluster separation and compactness (CH indices for k = 2,3…,10: [38.944, 37.938, 32.598, 27.206, 25.027, 22.893, 19.781, 20.022, 21.197]).

#### Assessing the contribution of different sound features to naïve perceptual similarity in individual humans

To estimate how strongly the three features of pulsed sound stimuli considered in our study affect their naïve perception in humans, we conducted a multiple linear regression, fitting the rated pairwise similarities from our scaling paradigm with a linear combination of the differences in the sound features. Before the regression, we pooled the data of all subjects. Specifically, we used all 190 unique pairwise similarity ratings of the 20 different pulsed sounds, obtained from the individual similarity matrices of the scaling paradigm (diagonal excluded), and transformed them into dissimilarity values with the operation $1-{similarity}$. Then, we fitted the 190 dissimilarity values of each individual with a linear combination of the differences in all three sound features (*pulses per second*, *irregularity of accents*, *pulse imbalance*) multiplied by a weight, using a least-squares algorithm with intersect. Thus, the regression expresses the perceptual dissimilarity of two given sounds $i$ and $j$ as $1-{{similarity}}_{{i\; vs}.{j}}=\,{{w}_{0}+w}_{1}\Delta {f}_{1}+{w}_{2}\Delta {f}_{2}+{w}_{3}\Delta {f}_{3}$, where $\Delta {f}_{x}={{|\, f}}_{x,{i}}-{f}_{y,j}|$ denotes the absolute difference in each of the three sound features between the sound pair, and ${w}_{0}$ denotes an offset to account for individual biases to generally respond with higher or lower similarity ratings. This approach assumes that pairs of sounds with highly distinct sound features will also be perceived differently. A high absolute weight for a given feature indicates that this feature strongly affects perceptual dissimilarities. Assumptions of linear regression for the pooled data were verified by inspection of residual normality (Q-Q plot), homoscedasticity (residuals vs. fitted), and absence of multicollinearity (variance inflation factor VIF = 1.123), confirming the suitability of the linear model. To systematically compare the impact of the different sound features on the perceptual similarities/dissimilarities, we computed the absolute contribution of each sound feature $x$ as the product of the respective weight and the mean feature difference of all unique sound pairs: ${contributio}{n}_{x}=|{w}_{x}\bar{\Delta {f}_{x}}|$. To further test the implications of different individual patterns of contributions on perceptual discrimination in our decision tasks, we correlated the contributions of each sound feature across all 152 participants with the estimated learning features and psychometric curve properties.

#### Consistency of individual to average perceptual maps

In order to quantify how strongly each participants individual perception of pulsed sounds was aligned to the general perceptual patterns of other participants, we computed the correlation of all off-diagonal elements of each individual similarity matrix in each condition (human scaling paradigm, human discrimination task with *pulses per second*, *irregularity of accents*, and *pulse imbalance*) to the mean similarity matrix obtained from averaging the data of all other participants. We used a t-test to compare the distributions of the task-based correlations with the consistency estimates from the similarity ratings.

#### Comparing average perceptual maps across modalities and species

Based on the similarity matrices in all conditions, we compared the structure of mean perceptual maps across the different discrimination tasks and across species by computing the Pearson correlation between all non-redundant off-diagonal elements of the mean similarity matrices in the respective condition. For display purposes only, we conducted a principal component analysis over each mean similarity matrix and plotted the similarity patterns of all sounds in the dimension-reduced space of the first two principal components.

In addition, for our human sample, we used the *mean off-diagonal similarity* as a measure to compare the aggregate perceived similarity across all stimuli and task conditions to different personality traits. The *mean off-diagonal similarity* was computed as the mean value of all 190 unique off-diagonal elements of the similarity matrix of each participant.

#### Predicting task-based perceptual maps from task-naïve perceptual maps

To test if the task-naïve perception of similarities between pulsed sounds in our scaling paradigm allowed to predict the following discrimination patterns between the same sounds in our perceptual discrimination tasks, we applied a multiple linear regression. Here, we modeled each individual’s task-based similarity estimates in each task modality (*pulses per second*, *irregularity of accents*, *pulse imbalance*) with a linear combination of this individual’s task-naïve similarity ratings, obtained from the scaling paradigm. Specifically, we iteratively went through all 190 unique off-diagonal elements of each individual’s similarity matrix in each of the three task modalities, and predicted each element $i$ – expressing a task-based similarity value of a given pair of sounds – from a linear combination of the naïve similarity ratings of all other 189 sound pairs with an intercept: ${{taskSim}}_{i}=\,{{w}_{0}+w}_{1}{naiveSi}{m}_{1}+{w}_{2}{naiveSi}{m}_{2}+{\ldots +w}_{190}{naiveSi}{m}_{190}$. The weights for this prediction were obtained from a regression of the respective sound pair data of all remaining 151 participants, using a least squares algorithm. This procedure allowed to obtain modeled behavioral similarity matrices for each individual and task modality, using this individual’s naïve similarity ratings as predictor, only. To compare the predictability of the observed data with random perceptual patterns, we conducted an equivalent regression with the training data shuffled over all sound-pairs for each participant independently. We then compared the goodness of the regression models by quantifying the mean squared error of each participant’s empirically observed task-based similarity matrix and the similarity matrices predicted from the regression models trained on the observed and shuffled data, respectively. This procedure was repeated for each task modality (*pulses per second*, *irregularity of accents*, *pulse imbalance*).

#### Logistic regression to assess perceptual decision strategies over the course of learning in mice

To test the individual influence of different sound features and stimulus-independent features on the perceptual decision behavior of mice over the course of learning, we applied a logistic regression model. For the model, we first separated all trials of each mouse into evenly sized, non-overlapping bins of 200 trials. Then, to assess the evolution of decision strategies over the course of learning, we used the 200 trials of each bin to independently apply the following regression: We modeled the individual probability of mice to poke in a given trial of the task $t$ with the logistic function ${p}_{{poke}}=\frac{1}{1+{e}^{-\theta }}$, where $\theta ={w}_{{bias}}+{w}_{{inertia}}{prevPoke}+{w}_{1}\Delta {f}_{1}+{w}_{2}\Delta {f}_{2}+{w}_{3}\Delta {f}_{3}$. In this equation, θ expresses a linear combination of different regression parameters and their corresponding weights that independently impact on choice behavior. *Bias*: ${w}_{{bias}}$ depicts a stimulus-independent bias towards a poking response^44,45^. *Inertia*: ${w}_{{inertia}}{prevPoke}$ depicts a stimulus-independent tendency to repeat the previous choice^46–48^, where ${prevPoke}$ takes either a value of +1 if the response in trial $t-1$ was a poke, or a value of -1 if the previous response was no poke. *Sound features*: ${w}_{{{\mathrm{1,2,3}}}}\Delta {f}_{{{\mathrm{1,2,3}}}}$ express stimulus-dependent effects of the three features *pulses per second* (denoted by index 1), *irregularity of accents* (denoted by index 2) and *pulse imbalance* (denoted by index 3) of the pulsed sound in the current trial. For each respective sound feature, $\Delta {f}_{{{\mathrm{1,2,3}}}}$ describes the difference of the currently sampled sound to all pulsed sounds with 11 pulses per second (clearly assigned as S + ) relative to all sounds with 3 pulses per second (clearly assigned as S-). Specifically, for the exemplary feature *pulses per second* this relative difference was computed as $\Delta {f}_{1}=\,\bar{\Delta {f}_{1,S-}}-\bar{\Delta {f}_{1,S+}}$, leading to high values in the case of high relative similarity to the reinforced stimuli with 11 pulses, or to a low value in the case of high relative similarity to punished stimuli with 3 pulses. The relative difference in the other features was computed analogously. We fitted the weights of all parameters in the regression over all trials in each respective bin, using a maximum likelihood approach. As a metric of model goodness, we then evaluated the fraction of correctly predicted trials from the fitted regression model. To compare the effects of the different regression features, we estimated the contribution of each regression parameter on individual decision behavior by considering the weights normalized by the respective parameter values. In particular, we computed the contribution of each sound feature $x$ as ${contributio}{n}_{x}=\overline{|{w}_{x}\Delta {f}_{x}|}$. In a next step, we normalized the contribution of each parameter to the sum of all parameter contributions in the current bin, expressing it as a fraction of impact on choice relative to all other model parameters. Using the parameter contributions as a proxy for decision strategies in perceptual choice tasks, we further tested how the dynamics of parameter contributions over the course of learning related to learning success and rewards. For this purpose, we pooled the contributions of each parameter over trial bins and individuals, and computed the Pearson correlation to the pooled average performance of each bin and individual. We applied a Bonferroni-Holm correction to all reported p-values for all regression parameters.

#### Logistic regression to assess perceptual decision strategies over the course of learning in humans

To test the individual influence of different sound features and stimulus-independent features on the perceptual decision behavior of humans, we followed a logistic regression approach, analogous to the analysis of our mouse data. We chunked the data of each task condition into five evenly-sized bins of 34 trials. For each bin, we applied an independent regression analysis, where we modeled the probability of a response to the reference alternative ${p}_{{resp}}=\frac{1}{1+{e}^{-\theta }}$, where $\theta ={w}_{{bias}}+{w}_{{inertia}}{wasPoke}+{w}_{1}\Delta {f}_{1}+{w}_{2}\Delta {f}_{2}+{w}_{3}\Delta {f}_{3}$. The regression specifics followed our mouse analyses, where the relative difference in each feature value $\Delta {f}_{{{\mathrm{1,2,3}}}}$ was computed relative to all 8 stimuli assigned to the reference alternative and the other alternative (e.g. $\Delta {f}_{1}=\,\bar{\Delta {f}_{1,{ref}}}-\bar{\Delta {f}_{1,{nonRef}}}$).

### Reporting summary

Further information on research design is available in the Nature Portfolio Reporting Summary linked to this article.

## Results

### Stimulus features are encoded to a varying degree in naïve perceptual maps

**Fig. 1:**
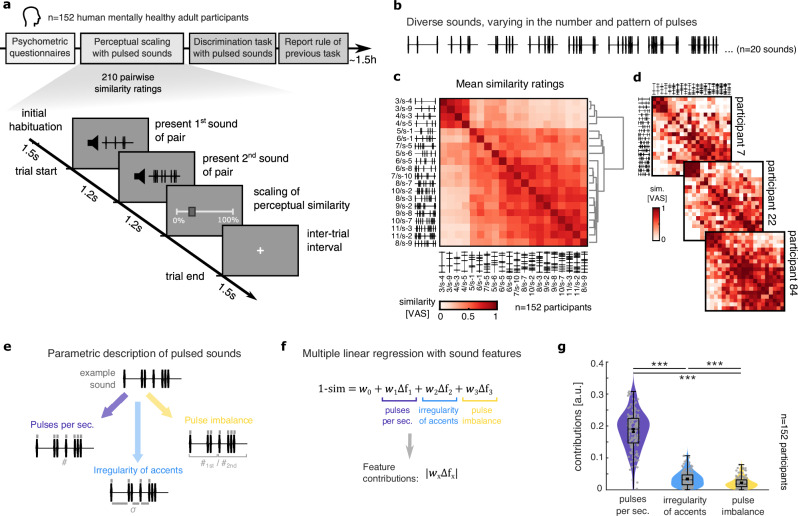
Stimulus features of pulsed sounds are encoded to a varying degree in naïve perceptual maps. **a** Flow of human experiment, including a perceptual scaling paradigm to assess task-naïve perceptual maps by rating the similarities between pairs of pulsed sounds on a visual analog scale (VAS). **b** A library of 20 different sounds was used, varying in the pulse number and temporal pattern (for details see Methods and Supplementary Fig. 1). **c** Mean matrix of all pairwise similarity ratings over all 152 participants, serving as proxy of an average naïve perceptual map. The stimuli are sorted by the dendrogram of their similarity patterns (see right side). This sorting of stimuli is applied to all similarity matrices throughout this manuscript to allow comparability of map structures. **d** Perceptual map estimates of single example participants, depicting a diversity in individual similarity ratings. **e** The pulsed sound stimuli were described by three features – *pulses per second*, *irregularity of accents*, *pulse imbalance* – independently capturing their energy and temporal structure (see Methods). **f** Linear regression to fit each participant’s similarity ratings with a combination of the differences in stimulus features and estimate the *contribution* of each stimulus feature on map structure. **g** Contributions on task-naïve perceptual maps, showing a dominant impact of *pulses per second* (grey dots represent 152 single individuals; thin lines indicate the median, the quartiles around the median, and the whiskers indicate top and bottom quartiles; thick black bar indicates the mean ± SEM; ***p < 0.001 in Wilcoxon signed-rank test).

In our study, we sought to investigate and compare the structures of task-naïve and task-based perceptual maps for a fixed set of complex sound stimuli. For this purpose, we recruited a sample of 152 students free from active hearing impairment and neuropsychiatric disorders (demographics detailed in Supplementary Table 1), and devised a paradigm in which participants first underwent a reinforcement-free scaling task with a set of pulsed sound stimuli, and subsequently completed choice tasks to discriminate the same set of stimuli under three different reinforcement rules (Fig. 1a; Methods). Note, that we refer in this study to participants that have not yet been involved in the reinforcement-based discrimination task as *task-naïve*.

Parts of the scaling data have been previously analyzed to address a question unrelated to the current study (see Methods and ref. ^22^). The one-second-long pulsed sound stimuli consisted of a sequence of brief white noise bursts, and systematically varied in their number of pulses (ranging from 3/s up to 11/s) as well as in their temporal pulse pattern providing a stimulus set essentially free from inherent semantic meaning (Fig. 1b, Methods, Supplementary Fig. 1a).

First, we investigated perceptual maps in task-naïve conditions free from reinforcement, using the scaling task, in which participants scored all pairwise similarities between a set of 20 different pulsed sounds. Specifically, participants underwent 210 trials, each of which comprised the presentation of a given pair of sounds with the subsequent instruction to rate the degree of similarity between the sampled sounds on a visual analog scale (see Methods for task details). Note that the order of stimuli was randomized to avoid confounding of the perceptual ratings by sequence biases. These pairwise similarity ratings allowed us to construct a 20-by-20 similarity matrix for each participant which we further used as a proxy of an individual’s perceptual map (Methods). Building the mean similarity matrix across participants, we observed two dominant similarity clusters – one with sounds containing ≤4 pulses per second, and one containing ≥5 (Fig. 1c; optimal partitioning of the data in k = 2 clusters with Calinski-Harabasz index = 38.944, see Methods). Individual similarity matrices largely mirrored this average map structure, however also exhibited marked inter-individual differences (Fig. 1d).

The structure of a perceptual map is determined by the extent to which a given property of a stimulus is encoded. To unravel the relative contribution of different stimulus properties on the map structure, we first parametrized all sounds according to a set of three qualitatively different stimulus features, capturing cumulated stimulus energy as well as temporal features, such as pulse regularity and the temporal modulation of pulse density (Fig. 1e, Methods). Specifically, we quantified the number of *pulses per second* (short: pulses), the *irregularity of pulse accents* (short: irregularity) and the *imbalance of pulses* (short: imbalance) for all pulsed sounds in our library (Supplementary Fig. 1b–d). These sound features have been shown to be prominently encoded in the mouse auditory cortex^23^, conforming to previous studies that investigated the auditory representation of similar features^49–51^.

To estimate the relative extent to which the three stimulus features determine the perceived similarity or dissimilarity between a pair of sounds, we conducted a linear regression. We modeled each participant’s similarity ratings based on the differences in stimulus features of the two sounds, allowing us to estimate the impact of the different features on the structure of individual naïve perceptual maps (Fig. 1f, Methods). We observed that *pulses per second* dominated perceptual map structure, followed by *irregularity of accents* and *pulse imbalance* (Fig. 1g; *n* = 152 participants, mean contribution ±SD of: pulses 0.186 ± 0.060, irregularity 0.034 ± 0.023, imbalance 0.022 ± 0.017; Wilcoxon signed rank test, *n* = 152 participants: pulses vs. irregularity z = 10.685, *p* < 0.001, pulses vs. imbalance z = 10.689, *p* < 0.001, irregularity vs. imbalance z = 4.444, *p *< 0.001). To test if the features in our stimulus set show systematic inherent interactions, we correlated them with each other, finding no statistically significant effect (Supplementary Fig. 1e; *n* = 20 sounds, pulses vs. irregularity: R_P_ = -0.344, CI = [−0.683, 0.116], *p* = 0.138, pulses vs. imbalance: R_P_ = 0.212, CI = [−0.254, 0.598], *p* = 0.370, irregularity vs. imbalance: R_P_ = 0.397, CI = [−0.055, 0.714], *p* = 0.083). Our observation of marked differences in the contributions indicate that under basal, task-naïve conditions the degree of representation in perceptual maps varies substantially across the three sound features.

### Humans learn to discriminate different features of pulsed sounds with varying ease

**Fig. 2:**
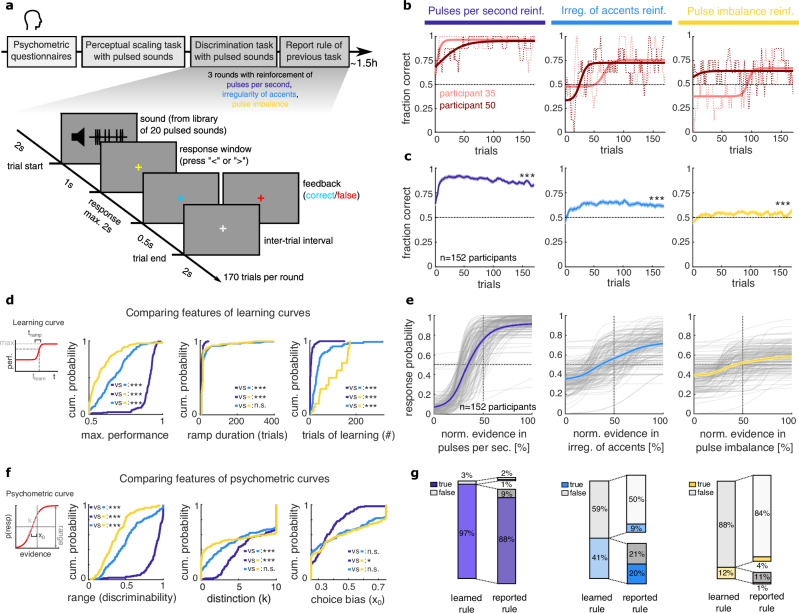
Humans learn to discriminate different features of pulsed sounds with varying ease. **a** The human experiment further included three discrimination tasks to probe task-based perceptual maps under different discrimination rules (see Methods for details). **b** Learning curves of two example subjects for all three task conditions (dotted line: raw data; solid line: fitted learning curve; see Methods). **c** Mean learning curves over all participants (*n* = 152; shaded area depicts SEM; ***p < 0.001 in a *t*-test comparing the mean performance in trials 86–170 against chance level). The slight decline of mean performance over time is due to a gradually increasing probability to present ambiguous stimuli (see Methods). Data represented as mean ± SEM. **d** Cumulative distributions of learning curve parameters across task conditions. Individual learning curves were fitted with a sigmoid to assess the maximal performance, the number of trials for performance to ramp to a plateau, and the total number of trials until reaching a performance of 70% (Methods; *n* = 152 participants; ***p < 0.001 in a Wilcoxon rank sum test). **e** Psychometric curves of stimulus discrimination in all task conditions (x-axis depicts normalized values in each sound feature; *n* = 152 participants; grey lines: single individuals, strong line: mean, shaded area: ±SEM). **f** Cumulative distributions of psychometric curve parameters in all task conditions. Individual psychometric curves were estimated by a sigmoid to quantify the maximal discriminability (range), the clarity of distinction (**k**), and the response bias (x_0_) toward high values in each sound feature (*n *= 152 participants per condition; ***p < 0.001, *p < 0.05 in a Wilcoxon rank sum test; see Methods). **g** Fractions of participants that successfully learned the discrimination tasks (reaching a performance ≥70%), and reported the correct rule of the tasks.

We next probed perceptual maps under task-based conditions, using a discrimination task with varying reinforcement rules. All participants completed three rounds of a two-alternative forced choice task, each of which assigned reinforcements based on one of the three different stimulus features (*pulses per second*, *irregularity of accents*, *pulse imbalance*), so that participants had to discriminate the same set of sounds, but according to varying rules (Fig. 2a, Methods). Participants were instructed that each task had the same structure, but independent rules. This procedure allowed us to compare the perceptual discrimination of the same stimuli according to different, qualitatively distinct features, providing independent estimates of task-based perceptual maps for each task condition. Each task comprised 170 trials during which participants had to learn to discriminate the pulsed sounds based on the feedback (correct/false) provided after each choice, which entailed to estimate the reinforced stimulus feature and its decision criterion. Following each discrimination task, participants were asked to report their inferred rule for the preceding task.

Across subjects, performance improved in all task conditions, albeit to varying degrees (Fig. 2b). At the group level, average performance was high for *pulses per second*, intermediate for *irregularity of accents* and was barely above chance for *pulse imbalance* (Fig. 2c; *n* = 152 participants, mean performance in 2nd half ±SD: pulses 86 ± 11%, irregularity 63 ± 14%, imbalance 54 ± 10%; *t*-test performance in 2nd half vs. 0.5, *n* = 152 participants: pulses *t*(151) = 40.556, *p* < 0.001, irregularity t(151) = 12.191, *p *< 0.001, imbalance t(151) = 4.753, *p* < 0.001). To quantify learning dynamics, we fitted sigmoid curves to each participant’s data and compared (i) maximal performance, (ii) learning curve steepness (ramp duration), and (iii) number of trials to reach a performance ≥70% correct choices (Fig. 2d, see Methods). Reinforcing *pulses per second* yielded the highest and fastest learning (mean ± SEM, *n* = 152 participants, max. performance 90 ± 8%, ramp duration 10 ± 6, trials to learning 6 ± 14), significantly exceeding learning in the other tasks (*n* = 152 participants, Wilcoxon rank-sum test: *p* < 0.001 in all comparisons). Reinforcement of *irregularity of accents* resulted in intermediate learning (max. performance 67 ± 12%, ramp duration 35 ± 87, trials 43 ± 53), while learning induced by reinforcement of *pulse imbalance* was lowest and slowest (max. performance 59 ± 11%, ramp duration 32 ± 103, trials 98 ± 59) (Wilcoxon rank-sum test, *n* = 152 participants, max. performance: pulses vs. irregularity z = 13.121, p < 0.001, pulses vs. imbalance z = 13.503, *p* < 0.001, irregularity vs. imbalance: z = 6.844, *p* < 0.001; ramp duration: pulses vs. irregularity z = −7.599, *p* < 0.001, pulses vs. imbalance z = −7.289, *p *< 0.001, irregularity vs. imbalance: z = 0.594, *p* = 0.553; trials: pulses vs. irregularity z = −10.310, *p* < 0.001, pulses vs. imbalance z = −7.309, *p* < 0.001, irregularity vs. imbalance: z = −3.819, *p* < 0.001). Thus, participants successfully learned to discriminate pulsed sounds according to different independent features, where discrimination along stimulus energy was learned faster and more efficiently as compared to temporal stimulus patterns. In a next step, we sought to quantitatively compare the goodness in perceptual discrimination across all sound features. For this purpose, we fitted psychometric curves for each participant and described the perceptual performance using different parameters capturing the maximal response contrast (*range*), the distinction of sounds along a given feature (*k*), and general choice biases to a given feature (*bias*) (Fig. 2e, see Methods). Mirroring the observed learning dynamics, we found that *pulses per second* was discriminated best (Fig. 2f; Wilcoxon rank sum test, *n* = 152 participants, range: pulses vs. irregularity z = 11.212, *p* < 0.001, pulses vs. imbalance z = 13.818, *p* < 0.001; k: pulses vs. irregularity z = 4.243, *p* < 0.001, pulses vs. imbalance z = 4.642, *p* < 0.001).

*Irregularity of accents* showed intermediate levels of discrimination, with clearer behavioral responses than *pulse irregularity* (Wilcoxon rank sum, *n* = 152 participants, range: z = 5.822, *p* < 0.001), but without statistically significant differences in distinction (Wilcoxon rank sum test, *n* = 152 participants, k: z = 1.602, *p* = 0.109). For all features, we observed a response bias towards higher feature values, that however was highest for *pulses per second* (Wilcoxon rank sum test, *n* = 152 participants, bias: pulses vs. irregularity z = −1.854, *p* = 0.064, pulses vs. imbalance z = −2.549, *p* = 0.011, irregularity vs. imbalance z = −0.683, *p* = 0.494). Together, these analyses demonstrate that the perceptual discriminability of pulsed sounds varies across different features and correlates positively with learning speed.

### Behavioral tasks can be learned independent from explicit insight

Based on the varying performances in discriminating pulsed sounds along different stimulus features, we wondered in how far participants explicitly understood the respective discrimination rules of the tasks. For this purpose, we compared the implicit behavioral learning, indicated by performances ≥70%, to the verbal estimations of the reinforcement rules after completion of the discrimination tasks (see Methods). While the general rates of explicit task insight matched the overall discriminability of sound features, we found a considerable mismatch between the implicit behavioral and explicit understanding of the task (Fig. 2g; fractions of participants exhibiting implicit behavioral vs. explicit understanding: *n* = 152 participants, pulses 97% vs. 89%, irregularity 41% vs. 29%, imbalance 12% vs 5%). The rate of implicit behavioral understanding of the task without corresponding explicit understanding increased from *pulses per second* (9 of 97%) over *irregularity of accents* (21 of 41%) up to *pulse imbalance* (11 of 12%). Conversely, a smaller fraction of subjects did not reach the behavioral learning criterion, but nevertheless reported the rule of the task correctly (pulses 1 of 3%, irregularity 9 of 59%, imbalance 4 of 88%). Together, these observations indicate a substantial mismatch between the behavioral discrimination and the explicit verbal report of the task rule for the more difficult tasks, consistent with ideas of divergent representations of implicit and explicit knowledge^52,53^.

### Individual discrimination performance is partially predicted by naïve perceptual maps

**Fig. 3:**
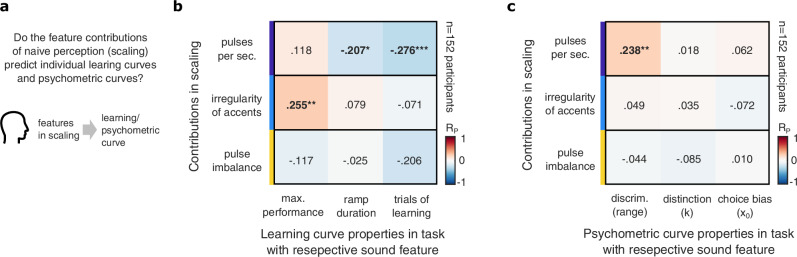
Individual discrimination performance is partially predicted by naïve perceptual maps. **a** We tested how the contributions of stimulus features to individual perceptual maps predicted the subsequent task-based discrimination of participants. **b** We correlated the individual feature contributions to naïve map structure (see Fig. 1g) with the individual learning curve features in the respective condition of the discrimination task (see Fig. 2d). **c** Equivalent correlation analysis with the individual psychometric curve properties (see Fig. 2f). The correlation matrices in b and c indicate significant predictability of some learning features and psychometric features based on the naïve perceptual contribution of *pulses per second* and *irregularity of accents*. (numbers indicate Pearson correlations; n = 152 participants; *p < 0.05, **p < 0.01, ***p < 0.001; bold font indicates *p* < 0.05).

Our analyses so far revealed that the degree of representation for the three stimulus features was highly similar in estimates of perceptual maps in naïve and task-based contexts. To further investigate in how far the structure of the two perceptual maps obtained in individual participants are related, we tested if learning performance can be predicted from naïve perceptual maps. We correlated the individual contributions of each stimulus feature to naïve perception with the learning properties and psychometric curve properties in the respective discrimination task condition (Fig. 3a). With this analysis, we tested if participants whose naïve perception was highly dominated by a given sound feature could also discriminate this feature better in the task, compared to participants with a lower perceptual contribution of this feature. Interestingly, we observed that indeed the naïve perceptual weights of *pulses per second* predicted significantly faster learning and steeper learning curves in the corresponding discrimination task (Fig. 3b; *n *= 152 participants; pulses vs. max. perf. R_P_ = 0.118, CI = [-0.042, 0.272], *p* = 0.148, pulses vs. ramp duration R_P_ = -0.207, CI = [−0.355, −0.049], *p* = 0.011, pulses vs. learning time R_P_ = -0.276, CI = [-0.417, −0.122], *p* = 0.001). The perceptual weights of *irregularity of accents* were positively correlated to maximal task performance (irregularity vs. max. perf. R_P_ = 0.255, CI = [0.100, 0.398], *p* = 0.002, irregularity vs. ramp duration R_P_ = 0.079, CI = [-0.081, 0.235], *p* = 0.335, irregularity vs. learning time R_P_ = −0.071, CI = [−0.228, 0.089], *p* = 0.572), whereas we observed no significant evidence for an association of *pulse imbalance* with learning (imbalance vs. max. perf. R_P_ = −0.117, CI = [−0.271, 0.043], *p *= 0.153, imbalance vs. ramp duration R_P_ = -0.025, CI = [−0.184, 0.135], *p* = 0.770, imbalance vs. learning time R_P_ = −0.206, CI = [−0.354, -0.048], *p* = 0.384). Considering the properties of psychometric curves, we observed a significant interaction between the naïve perceptual weights of *pulses per second* and the maximal discriminability (Fig. 3c; *n* = 152 participants; pulses vs. range R_P_ = 0.238, CI = [0.082, 0.383], *p* = 0.003, pulses vs. k R_P_ = 0.018, CI = [−0.142, 0.177], *p* = 0.831, pulses vs. x_0_ R_P_ = 0.062, CI = [−0.098, 0.219], *p* = 0.448), but did not find significant correlations of the other feature contributions with psychometric curve properties (irregularity vs. range R_P_ = 0.049, CI = [-0.111, 0.207], *p* = 0.549, irregularity vs. k R_P_ = 0.035, CI = [−0.125, 0.193], *p* = 0.671, irregularity vs. x_0_ R_P_ = -0.072, CI = [-0.229, 0.088], *p* = 0.380; imbalance vs. range R_P_ = -0.044, CI = [−0.116, 0.202], *p* = 0.587, imbalance vs. k R_P_ = -0.085, CI = [−0.241, 0.075], *p *= 0.298, imbalance vs. x_0_ R_P_ = 0.010, CI = [−0.149, 0.169], *p* = 0.903). This indicates that individual differences in task-based perceptual discrimination of pulsed sounds are partially predicted by differences in the impact of different features on naïve perception.

### Shared structural features of naïve and task-based perceptual maps

**Fig. 4:**
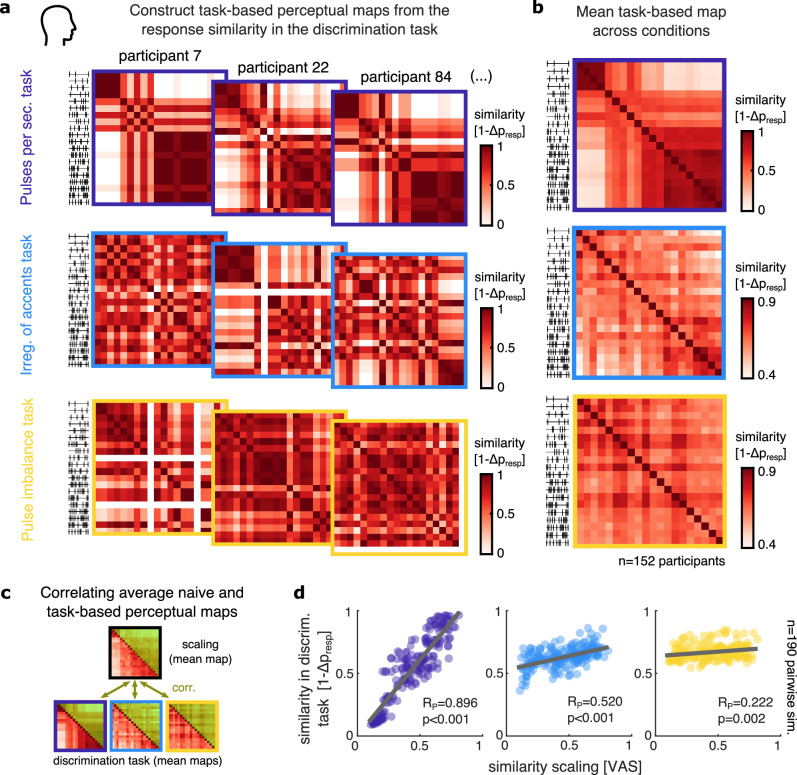
Shared structural features of naïve and task-based perceptual maps. **a** To compare the naïve and task-based perceptual map patterns across all pulsed stimuli, we estimated behavioral similarity matrices from the discrimination task data in each condition (computed as 1 minus the difference in response probabilities between each sound pair). The sorting of stimuli in all matrices of this Figure is equivalent to Fig. 1. Estimated similarity matrices from example participants are shown (entirely blank rows and columns represent sounds that were by chance not presented to the participant in the respective condition). **b** Mean similarity matrices obtained from the different discrimination tasks. **c** Comparison of the mean task-based map structure in each condition and the mean naïve perceptual map (equivalent to Fig. 1c). Note that only non-redundant off-diagonal elements are considered for this comparison via correlation (highlighted in green). **d** Pearson correlations of the mean map structures (*n* = 190 off-diagonal similarity values per correlation) indicating significant similarity between naïve map structure and task-based map structures.

While our previous analyses estimating the contribution of stimulus features to model perceived stimulus similarities provide a general and interpretable perspective on the architecture of a perceptual map (see Fig. 1), fine-grained information of the map structure obtained from the individual pairwise stimulus relations are not captured. In the next step, we explored the shared structural variability between naïve and task-based perceptual maps in more detail. For this purpose, we used the similarity matrices constructed from the data from the similarity ratings as proxies of individual, task-naïve perceptual maps and additionally computed three independent estimates of task-based perceptual maps: For each pair of sounds, we computed the difference in mean response probabilities over all trials in the task, and used the complement of this metric as a measure of task-based perceptual similarity (see Methods). This provided us with independent similarity matrices for each participant and task condition, respectively, showing marked variability across conditions and individuals (Fig. 4a). Based on these individual map estimates, we computed the mean task-based similarity matrices for all task conditions (Fig. 4b), providing proxies of average perceptual maps. The structure of the average and individual map estimates from the easy discrimination task (*pulses per second*) was pronounced, whereas it was less clear in the two estimates obtained from the more difficult tasks (*irregularity of accents*, *pulse imbalance*) (Supplementary Fig. 2a, b).

Next, we compared the average structures of task-naïve and task-based perceptual maps by correlating the mean similarity matrices from the discrimination tasks to the mean similarity matrix obtained from the scaling data (Fig. 4e, f). Here, we observed a high similarity between the structure of naïve perceptual map and the task-based map of *pulses per second* (*n* = 190 off-diagonal similarities, R_P_ = 0.896, CI = [0.864, 0.921], *p* < 0.001). Interestingly, although not apparent from the average similarity estimates (see Fig. 4d), also the task-based map estimates of *irregularity of accents* and *pulse imbalance* were significantly correlated to the naïve perceptual map (*n* = 190 off-diagonal similarities, irregularity: R_P_ = 0.520, CI = [0.408, 0.617], *p* < 0.001, imbalance: R_P_ = 0.222, CI = [0.082, 0.353], *p* = 0.002).

In an additional step, we tested if naïve perceptual maps could even predict the structures of task-based maps on an individual level. We found that a regression model trained on individual task-naïve perceptual maps could provide a moderate, but significant prediction how similar a particular stimulus pair would be perceived in the task context (Supplementary Fig. 2c, d). Moreover, we quantified the mean off-diagonal similarity across the perceptual similarity matrices for each participant, providing a measure of the average structure of task-based perceptual maps, and correlated this metric to the psychometric questionnaires in our study. We did not detect any statistically significant correlation between task-based perceptual map structures and personality characteristics or mental health indicators (Supplementary Fig. 2e).

Together, these observations indicate that the naïve perception of pulsed sound stimuli captures qualitatively different stimulus features, predicting the pairwise perceptual discrimination of these sounds under task-based conditions.

### Structures of perceptual maps are shared between humans and mice

**Fig. 5:**
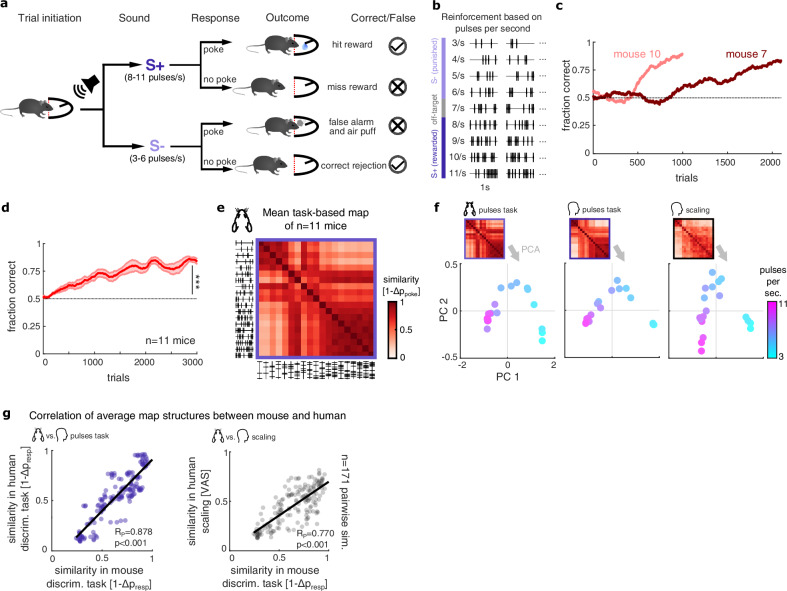
Structures of perceptual maps are shared between humans and mice. **a** Schematic of an auditory-cued go/no-go task, conceptually analogous to the human discrimination task (see Fig. 2a), in which *n* = 11 mice learned to discriminate the number of pulses per second of pulsed sound stimuli (see Methods for details). **b** An extended stimulus set of 90 pulsed sounds was used (see Supplementary Fig. 3; 3-6 pulses/s -> no-go cue (S-), 7 pulses/s -> no consequence, 8–11 pulses/s -> go cue (S + )). **c** Learning curves of two example mice with different learning speed. **d** Mean learning curve of all mice (*n *= 11; shaded area depicts SEM; ***p < 0.001 in a t-test of mean performance in trials 1500-3000 against chance level, data represented as mean ± SEM). **e** Mean response similarity matrix for all sounds shared between the mouse and human experiments, computed in analogy to Fig. 4b (*n* = 11 mice; Methods; Equivalent sorting of stimuli as in Fig. 1). **f** Dimension-reduced projection of the mean map structures of mice and humans using principal component analysis (PCA) (see Methods). Each dot represents a sound, and its color represents its respective number of *pulses per second*. **g** Left: Correlation between the mean task-based maps in mice and humans (only off-diagonal elements are considered, *n* = 171 similarity estimates). Right: Correlation between the mean task-based map in mice and the mean naïve perceptual map in humans (computed over *n* = 171 off-diagonal similarity estimates). Both analyses show a strong coherence of map structures across species (computed over *n* = 171 off-diagonal similarity estimates).

To assess the generality of our findings across species, we compared our estimates of human task-naïve and task-based perceptual maps to analogous perceptual map estimates derived from mouse data. Specifically, we used data of 11 wildtype C57BL/6 J mice that were trained in an auditory-cued go/no-go task, structurally equivalent to our human task, where we reinforced the perceptually dominant feature of *pulses per second* (Fig. 5a, Methods, see ref. ^23^). In this task, mice were moderately deprived from water and trained to discriminate an extended, but structurally equivalent set of pulsed sounds in order to receive water rewards (Fig. 5b, Supplementary Fig. 3a, Methods). Congruent to our human analyses, we did not observe strong interactions between the stimulus features across the sounds in this experiments (*n* = 90 sounds, pulses vs. irregularity: R_P_ = −0.264, CI = [−0.447, −0.060], *p* = 0.012, pulses vs. imbalance: R_P_ = 0.058, CI = [−0.151, 0.262], *p* = 0.589, irregularity vs. imbalance: R_P_ = 0.146, CI = [−0.063, 0.343], *p* = 0.169, see Supplementary Fig. 3b). In line with the paradigm of our human experiment, sounds with 8-11 pulses/s indicated the availability of a reward upon poking, thus serving as a “go” signal (S + ). Sounds with 3-6 pulses/s indicated that mice should not poke in order to avoid an aversive air puff, thus serving as a “no-go” signal (S-). Ambivalent sounds with 7 pulses/s served as off-target stimuli without any punishment or reinforcement. Mice successfully learned to discriminate the pulsed sounds over a period a few hundred trials, while the task difficulty was gradually incremented by introducing more ambiguous stimuli with intermediate pulse numbers close to 7 (Fig. 5c, d; see Methods; t-test performance in 2nd half vs. 0.5, *n* = 11 mice: *t*(10) = 20.238, *p* < 0.001). These learning dynamics were comparable to prior studies, using an equivalent task with qualitatively different stimuli^41,42,51^. After reaching performance levels higher than 70% (see methods), mice exhibited gradually modulated response probabilities that generally increased with the feature *pulses per second*, but also differed substantially for stimuli with the same number of pulses^23^.

In analogy to our human analyses, we constructed task-based similarity matrices from the average poking probabilities in the auditory go/no-go task after mice had successfully learned the initial task. Specifically, we considered data from the 19 sounds that were shared across the mouse and the human experiment and that were presented with a sufficient number of repetitions (Fig. 5e; see Methods). We then compared the structure of the mean similarity matrix obtained from mouse data to the mean similarity matrices obtained from our human ratings and the discrimination task using *pulses per second* for reinforcement, considering the pairwise relations between the same 19 sounds (Fig. 5f). We first conducted a principal component analysis to visually inspect the perceptual structure across conditions. We observed a similar projection of the stimulus similarities that systematically reflected the dominant stimulus feature *pulses per second*. In addition, we quantified the correlation between the mouse and human matrices, showing high and significant positive correlations of the task-based mouse map to both, task-naïve and task-based perceptual maps (Fig. 5g; *n* = 171 off-diagonal similarities, mouse pulses task vs. human pulses task: R_P_ = 0.878, CI = [0.838, 0.908], *p* < 0.001, mouse pulses task vs. human scaling: R_P_ = 0.770, *p* < 0.001). This finding demonstrates that in order to guide perceptual decisions, mice employ perceptual map structures that are largely aligned to human perception within and outside of a task setting.

### Dynamic reweighting of stimulus features during successful learning

**Fig. 6:**
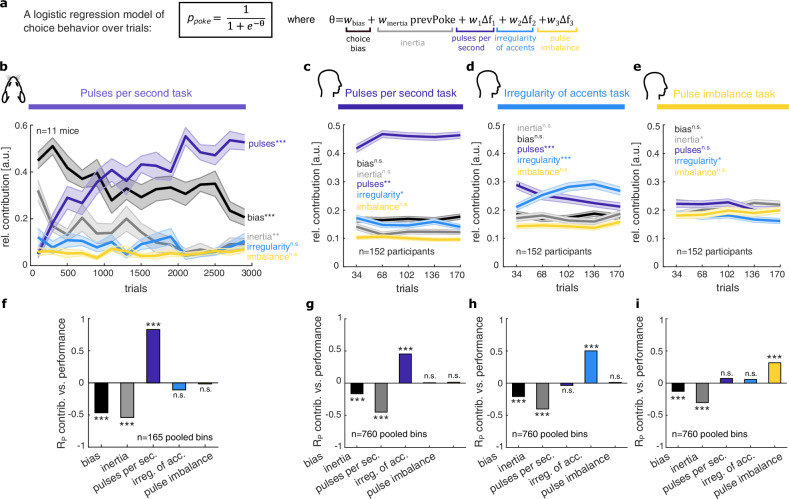
Increasing impact of reinforced stimulus features on perceptual decisions determines success of learning. **a** We applied a logistic regression to model each individual’s perceptual decision behavior based on the features of the experienced sounds (*pulses per second*, *irregularity of accents*, *pulse imbalance*) and stimulus-independent factors (*bias*, *inertia*). We chunked the data into independent bins over the course of learning and computed a regression for each bin, independently (see Methods). For mice, we modeled the poking probabilities in each trial, whereas for humans, we modeled the choice probability for one reference alternative (see Methods). **b** Relative contributions of the regression parameters in mice over the course of learning (see Methods; data represented as mean ± SEM). The reinforced stimulus feature (*pulses per second*) increased its contribution over the course of learning, whereas the unspecific factors (*bias* and *inertia*) decreased their contributions (*p < 0.05, **p < 0.01, ***p < 0.001 comparing the first and last bin in a *t*-test; *n *= 11 mice). **c–e** Equivalent analyses for the human data. **f** Pearson correlation of the relative contribution of each parameter in each time bin and the corresponding mean performance. As expected, decisions considering the reinforced feature, *pulses per second*, are associated with better task performance (R_P_ > 0, ***p < 0.001, *n* = 165 pooled bins), whereas decisions dominated by bias or inertia are associated with poorer performance (R_P_ < 0, **p < 0.01, ***p < 0.001, *n* = 165 pooled bins). **g–i** Equivalent analyses for the human discrimination tasks (*n *= 760 pooled bins).

Our observations support the notion that a largely static perceptual map is used as a basis for task-based behavior where learning can be achieved by adjusting the cognitive readout of this map. However, apart from stimulus-related, perceptual information also stimulus-independent factors can profoundly affect behavior ^41,44–48^. To shed light on the contribution of stimulus-related and non-stimulus related factors during the learning trajectory, we applied a logistic regression to describe the decision probability of each individual mouse and human (Fig. 6a, Methods). We modeled the poking/response probability, in each trial with a logistic function incorporating multiple parameters: *Stimulus-based* factors of perceptual decisions, describing the relative sensory dissimilarity of the sampled sound from the non-reinforced stimuli of the task, in respect to each of the sound features (*pulses per second*, *irregularity of accents*, *pulse imbalance*), as well as *stimulus-independent* regressors, namely a *bias* parameter, reflecting an idiosyncratic choice tendency^41,44,45^, and an *inertia* parameter, reflecting a general tendency to persist in previous choices^46–48^. In order to compare the effects of the regression parameters over the learning process, we then chunked the data of each individual into evenly sized trial bins over time, and modeled the response probabilities in each of the bins independently. From these regressions, we obtained independent estimates of the weight of each parameter over the course of learning, providing a proxy of how strongly each parameter contributed to the perceptual decision strategy of a subject.

As expected for a scenario of successful learning, we observed in our mouse data that the relative contribution of the reinforced sound feature, *pulses per second*, gradually increased over the course of learning, whereas the decision weights of the non-reinforced stimulus features did not change significantly (Fig. 6b; t-test of contributions in first vs. last trial bin, *n *= 11 mice: pulses t(10) = -11.509, *p* < 0.001, irregularity t(10) = 0.328, *p *= 0.750, imbalance t(10) = −0.431, *p* = 0.675) Interestingly, the contributions of the stimulus-independent factors *bias* and *inertia* were dominant at the start of training and decreased over the course of learning (t-test of contributions in first vs. last trial bin, *n* = 11 mice: bias t(10) = 5.500, *p* = 0.001, inertia t(10) = 3.741, *p* = 0.003), suggesting that initial behavior of mice was dominated by stimulus-independent choice factors that became suppressed over the course of learning. In contrast to mice, human behavior showed a comparably low susceptibility to stimulus-independent factors during any phase of the training. Human participants trained to discriminate stimuli according to *pulses per second* showed an increase in the weight of the reinforced stimulus feature (Fig. 6c; *t*-test of contributions in first vs. last trial bin, *n* = 152 participants: pulses t(151) = −2.865, *p* = 0.004), where however, stimulus-independent factors remained at a low impact on choice behavior, and only *irregularity of accents* exhibited a decreasing contribution (*t*-test of contributions in first vs. last trial bin, *n* = 152 participants: bias t(151) = -0.774, *p* = 0.440, inertia t(151) = 1.467, *p* = 0.145, irregularity t(151) = 2.382, *p* = 0.018, imbalance t(151) = 0.723, *p *= 0.467). This pattern was corroborated in the task where *irregularity of accents* served as reinforced feature. Here, we observed an increasing decision weight of the reinforced feature (Fig. 6d; *t*-test of contribution in first vs. last trial bin, *n* = 152 participants: irregularity t(151) = −3.458, *p* < 0.001) and a decreasing weight of pulses per second (*t*-test first vs. last trial bin, *n* = 152 participants: pulses t(151) = 4.363, *p *< 0.001), while the other features showed no significant weight dynamic (*t*-test first vs. last trial bin, *n* = 152 participants: bias t(151) = 0.733, *p* = 0.465, inertia t(151) = −1.065, *p* = 0.289, imbalance t(151) = −1.218, *p* = 0.225). In the discrimination task where *pulse imbalance* had to be discriminated, subjects also tended to increase their decision weight for this feature, however without reaching statistical significance (Fig. 6e; t-test first vs. last trial bin, *n* = 152 participants: imbalance t(151) = −1.255, *p* = 0.211). The other regression parameters showed unspecific dynamics, where the contribution of *inertia* to choice mildly increased, whereas the contribution of *irregularity of accents* slightly decreased over time (bias t(151) = 0.481, *p* = 0.631, inertia t(151) = −2.222, *p* = 0.028, pulses t(151) = 1.241, *p* = 0.216, irregularity t(151) = 2.070, *p* = 0.040). Importantly, for all regressions of our mouse and human data, we observed a largely stable degree of model goodness over the different trial bins (see Supplementary Fig. 4).

Furthermore, we tested how the different decision weights throughout learning related to differences in task performance by correlating the contributions of each parameter with the mean performance across bins (Fig. 6f–I; see Methods). For both species and all task conditions, we observed that higher weights of the reinforced stimulus features were associated with better performance, as expected (mice, *n* = 165 pooled bins: R_P_ = 0.830, CI = [0.776, 0.872], *p* < 0.001; human pulses, *n* = 760 pooled bins: R_P_ = 0.453, CI = [0.395, 0.508], *p* < 0.001; human irregularity: R_P_ = 0.501, CI = [0.446, 0.552], *p* < 0.001; human imbalance: R_P_ = 0.316, CI = [0.251, 0.379], *p* < 0.001). Additionally, the stimulus-independent features, *bias* and *inertia*, showed negative associations with task performance throughout, while non-reinforced stimulus-based features did not show significant interactions (mice, *n* = 165 pooled bins: bias: R_P_ = −0.400, CI = [−0.521, −0.263], *p* < 0.001, inertia: R_P_ = -0.539, CI = [−0.639, −0.421], *p* < 0.001, irregularity: R_P_ = -0.109, CI = [-0.258, −0.045], *p* = 0.326, imbalance: R_P_ = −0.016, CI = [−0.168, 0.137], *p* = 0.842; human, *n* = 760 pooled bins: pulses: bias R_P_ = −0.165, CI = [−0.233, -0.095], p < 0.001, inertia R_P_ = -0.451, CI = [-0.506, -0.393], p < 0.001, irregularity R_P_ = 0.005, CI = [-0.076, 0.066], *p* = 0.881, imbalance R_P_ = 0.014, CI = [-0.057, 0.085], *p* = 0.705; human irregularity: bias R_P_ = -0.208, CI = [−0.275, 0.139], *p* < 0.001, inertia R_P_ = −0.403, CI = [−0.461, -0.342], *p* < 0.001, pulses R_P_ = -0.037, CI = [-0.108, 0.034], *p* = 0.315, imbalance R_P_ = 0.011, CI = [−0.060, 0.082], *p* = 0.759; human imbalance: R_P_ = -0.128, CI = [−0.197, −0.057], *p* < 0.001, inertia R_P_ = −0.303, CI = [-0.366, -0.237], *p* < 0.001, pulses R_P_ = 0.073, CI = [0.002, 0.143], *p* = 0.088, irregularity R_P_ = 0.057, CI = [−0.014, 0.128], *p* = 0.114). Together, these observations indicate that mice and humans flexibly adapt their perceptual choices to the demands of a task at hand, where initial decisions in mice are dominated by stimulus-independent factors, whereas initial behavior in human primarily involves stimulus-dependent features that are re-adjusted over the course of learning.

## Discussion

In this study, we used psychophysics to characterize perceptual maps of pulsed sound stimuli in humans and mice. We estimated task-naïve perceptual structures and compared them to maps obtained during discrimination tasks with different rules. Sound features reflecting energy and temporal pattern contributed differently to naïve and task-based perception, with a dominant role of pulse number. Individual differences in naïve weighting of perceptual features predicted aspects of learning speed. Naïve perceptual maps enabled prediction of discrimination behavior at both population and individual levels. Perceptual structures also generalized across species, indicating a significant degree of conservation in map formation. Statistical modeling of learning dynamics showed that both humans and mice adapt decisions during learning by discounting unrewarded biases or features.

### Representation of stimulus features

To study sensory representations, we leveraged pulsed sounds differing only in their temporal structure, while sharing largely equivalent spectral properties, thus requiring temporal integration for their discrimination^24,25^. Moreover, these temporal modulations are deemed essential in determining the perceptual character of stimuli^54–58^, and are shown to be relevant for effective vocal communication in many species, including rodents and primates^59–64^, as well as for other sensory modalities, such as somatosensation^65–67^ or vision^24^. We used naïve perceptual ratings of stimulus similarity in humans under conditions without reinforcement (see Fig. 1) to construct a perceptual map that represented three stimulus features capturing acoustic energy (*pulses per second*) as well as temporal pattern (*irregularity of accents*, *pulse imbalance*) to a varying degree. This combination of features has been shown to be dominantly encoded at the level of the auditory cortex^51,68–71^, and is assumed to have a crucial role in auditory perception^24–28,72^. In our study, the feature *pulses per second* appeared to have a dominant influence on auditory perception, showing the highest impact on task-naïve ratings of perceptual similarity, whereas temporal stimulus features exhibited a significantly lower perceptual impact. These observations highlight the perceptual role of rate coding in the auditory system^70,71^, suggesting that one key mechanism to process auditory information is to integrate sensory signals over time and represent the integrated information in order to inform perception.

### Naïve perceptual maps predict task performance

We observed that humans exhibited consistent structures of perceptual maps between naïve conditions and task context, enabling us to predict task-based perceptual decisions from naïve ratings of stimulus similarity. We observed that the extent of representation of the three stimulus features in average naïve perceptual maps predicted the participants’ consecutive learning speed and final performance levels (see Fig. 2), indicating profound links between task-naïve and task-based perception. Learning abilities were correlated with explicit insight in the rules of the task, but a notable fraction of individuals also performed well in the task without explicit understanding. In the future, it will be interesting to assess task insight in greater detail, combining longitudinal assessments of understanding over the course of learning with specific instructions to facilitate or hinder this insight. Focusing on individual perceptual maps we furthermore found that variability in performance among participants in a particular discrimination task was reflected by the degree to which this stimulus feature was represented (see Fig. 3). Furthermore, we found a significant correlation in the fine structure of the individual perceptual maps under naïve and task-based conditions (see Fig. 4). These observations are not trivial and raise the question, how perceptual maps could support decision making in a task context. Two extreme scenarios may provide a reference: (i) A general representational map has formed before the task^73^ and behavior during the task depends on an optimized read-out procedure, leading to adjusted inferences from this otherwise stable representational map^74–76^. (ii) Learning during the task leads to the formation of a novel, separate representational map with an optimal configuration for the specific demands of the task^77–79^. These hypothetical extreme scenarios highlight that a correlation between task-based and task-naïve perception is not guaranteed. Given the psychophysical approach in our study without simultaneous recordings of neural activity, our dataset does not allow to entirely disentangle these two scenarios. Nevertheless, our findings that naïve perceptual maps show a high degree of correlation to task-based perceptual maps and task performance are consistent with the first scenario, assuming a context-dependent readout of a pre-existing representational map. There is growing evidence that such maps in the brain are formed and shaped by the statistics of prior experience^73,80^ and actively maintained^75,81^. It was shown in various studies that the representational relations between stimulus-evoked activity patterns are remarkably stable under basal conditions^76^ as well as during learning^8,67,82,83^. Consistent with the employment of a pre-existing map, we observed a fast and flexible adaptation of perceptual choice behavior to varying task contingencies in humans within comparably few trials. Theoretically, this observation could also result from rapidly restructured representational maps, however, in the light of the general tendency of stability in basic perceptual representations, such a strong remodeling appears unlikely^84^.

We did not observe a significant interaction of task-based map structures and basic personality traits. However, it was recently shown that humans who perceive a higher total degree of similarity between semantic or auditory stimuli also tend to think more creatively^22,85,86^. This observation suggests that perceptual similarity between different stimuli might be an important factor of their associability, so that individuals that form stronger perceptual links are more likely to develop innovative solutions. In future translational studies, detailed characterizations of perceptual maps may allow to investigate creativity mechanistically in the context of learning behavior, providing a potential bridge between human and non-human research^87^.

### Similar perceptual maps in humans and mice

We found that the perceptual maps in humans and mice showed a remarkable degree of similarity for the task-based similarity assessments as well as for the task-naïve ratings of stimulus similarity (see Fig. 5). Hence, the discrimination and categorization of pulsed sound stimuli appear to follow general perceptual structures that are largely similar across species, likely reflecting the high degree of similarity in the cell types and connectivity in mammalian brains. Moreover, our findings hint that fundamental temporal statistics of the acoustic environment in humans and mice are largely shared, driving the formation of similarly structured maps. Notably, the perceptual similarity across species was overt even though the human and mouse task were not entirely congruent and varied in their choice symmetry (two-alternative forced choice task in humans vs. go/no-go task in mice), consistent with a structure of the perceptual map independent from the specific task context.

Interestingly, it has been found that perceptual relatedness between different stimuli are encoded as the similarity of their corresponding neuronal activity patterns^18^, setting a starting point to link neural stimulus-processing with individual perception and shared behavioral features across species^10,25^. Using in vivo two-photon calcium imaging of population activity patterns in the auditory cortex of passively listening mice in response to the same set of pulsed sound stimuli, we have previously shown that the representational structure at the neuronal level captures entangled properties of the pulsed sounds described by the three stimulus features and reflects major aspects of the perceptual map^23^.

### Dynamic reweighting of stimulus features during learning

When modeling the learning dynamics in mice and humans, we observed that subjects increasingly used the reinforced perceptual features in their decisions over the course of learning. (see Fig. 6). Reward-unrelated features were concomitantly disregarded. Despite the similarities in learning dynamics in humans and mice, learning speed was markedly slower in mice. Our analysis revealed that initial mouse behavior was dominated by stimulus-independent choice factors. In contrast, initial human behavior was dominated by stimulus-related factors right from the beginning that, however, needed to be re-adjusted during learning to match the current reinforcement rule. Although the psychophysical approaches in humans and mice used in this study were largely comparable in structure, some differences in the task design were unavoidable. This implies that apart from the neurobiological differences between the species, causing mice to take much longer than humans to adjust their perceptual decisions and learn the discrimination, some methodological differences could also contribute.

A flexible re-mapping of behavioral responses to the different features represented on the same perceptual map could disentangle reinforced features from non-reinforced features according to the current demands of an individual, allowing for behavioral adaptation despite maintained value- and rule-related codes in higher cortical areas^46,88,89^. To allow this adaptation, perceptual learning likely includes on the one side, an optimization of the representational structures of a map to allow a quick readout, and on the other side, an optimization of readout strategies to couple sensory representations with different behaviors. Our observation that human participants show high discrimination performance despite lacking explicit insight in the rules of the task, supports a primary involvement of low-level perceptual representational maps rather than higher-level cognitive representational maps^52,53^.

### Limitations

Various limitations need to be considered for the interpretation of our study. Our human sample was limited to student participants and showed an uneven gender distribution, potentially reducing the generalizability of our findings to other populations. Hearing abilities were assessed via self-reports only. In addition, compared to our human sample undergoing perceptual discrimination tasks for three different stimulus features, our mouse cohort was substantially smaller and only underwent perceptual discrimination of one single stimulus feature. The tasks in humans were shorter than the mouse experiment, leading to an asymmetry in the number of trials considered in our analyses. Replication of our experiments, using a larger mouse sample and more extensive discrimination tasks of more stimulus features could in the future corroborate the initial observation in our cross-species comparison. Despite showing statistical significance, the correlations between map structures across task conditions and the correlation to learning abilities in the discrimination tasks often showed only intermediate or weak effect sizes, highlighting that a substantial degree of variance in perceptual map structures might be affected by e.g. finite sampling of pairwise similarities and additional biological factors, not considered in our study. Furthermore, our study design of assessing task-based perceptual maps under different conditions for the same stimuli does not allow to disentangle if the correlations between map structures fully result from shared representations, or from structural relations between the stimuli in our library. Even though, we did not find strong correlations between sound features in our stimulus set, future generalization studies with independent stimulus sets will be necessary to solve this issue. We primarily use linear models to quantify correlations or regression analyses in our data, finding that these comparably simple statistical approaches suffice to reveal significant effects. Future studies including additional non-linear models may in the future be used to resolve finer differences in the structures of perceptual maps.

## Conclusion

Taken together, our study demonstrates how the auditory perception of pulsed sound stimuli follows a general structure that is largely conserved across basal conditions, different task demands, and different species, serving as a substrate to inform and flexibly adjust perceptual decisions in changing environments.

## Supplementary information

Transparent Peer Review file

Supplemental Information

Reporting Summary

## Data Availability

The data of this study and the pulsed auditory stimuli used in this study have been deposited at G-Node (www.g-node.org) and is publicly available (10.12751/g-node.bgb999).

## Code availability

The MATLAB code used to analyze the data of this study has been deposited at G-Node (www.g-node.org) and is publicly available (10.12751/g-node.bgb999).
