# Supplementary material for: Auditory perceptual maps in humans and mice share common structures and predict perceptual decisions in discrimination learning
Source: Commun Psychol. 2026 Jun 20;4:98. doi: 10.1038/s44271-026-00485-w (PMC13282483; doi:10.1038/s44271-026-00485-w)
Supplement: Supplementary file 2 — Supplemental Information [file 44271_2026_485_MOESM2_ESM.pdf]

Auditory perceptual maps in humans and mice share common structures and predict perceptual decisions in discrimination learning

Johannes P.-H. Seiler[1], Giuseppe Cazzetta[1], Aida Ghobadi[1], Simon Rumpel[1]

[1] Institute of Physiology, Focus Program Translational Neurosciences, University Medical Center of the Johannes Gutenberg University Mainz, Duesbergweg 6, 55128 Mainz, Germany

Correspondence concerning this article should be addressed to Johannes Seiler or Simon Rumpel, Institute for Physiology, Focus Program Translational Neurosciences, University Medical Center of the Johannes Gutenberg University Mainz, Duesbergweg 6, 55128 Mainz, Germany. E-mail: johseile@uni-mainz.de, sirumpel@uni-mainz.de

- Supplementary information -

Supplementary Figures

Supplementary Figure 1

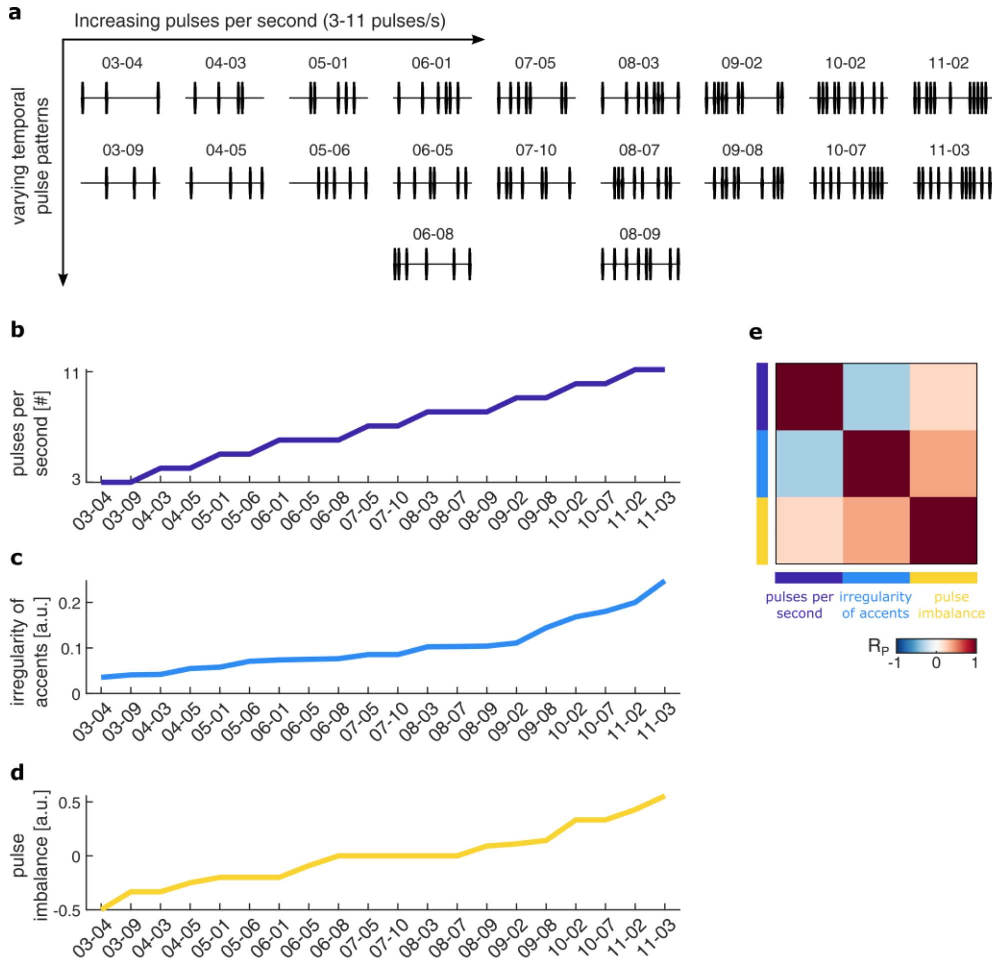

Supplementary Figure 1 – A set of pulsed sound stimuli to test perceptual discrimination in humans: (**a**) We used a set of 20 pulsed sounds covering different pulse categories (3-11 pulses/s) and different temporal patterns in a human discrimination task. (**b-d**) The selected sounds were characterized by three sound features (*pulses per second*, *irregularity of accents*, *pulse imbalance*) and were sorted by each of the sound features, corresponding to the reinforcement rules of the different tasks. The sounds showed a widely uniform distribution in all three features, suggesting comparable degrees of objective discriminability. (**e**) Correlation matrix of the three sound features for all 20 sounds. We did not find any significant correlation between the sound features (*pulses per second* vs. *irregularity of accents*: R_P=-0.344, p=0.138, *pulses per second* vs. *pulse imbalance*: R_P=0.212, p=0.370, *irregularity of accents* vs. *pulse imbalance*: R_P=0.397, p=0.083).

Supplementary Figure 2

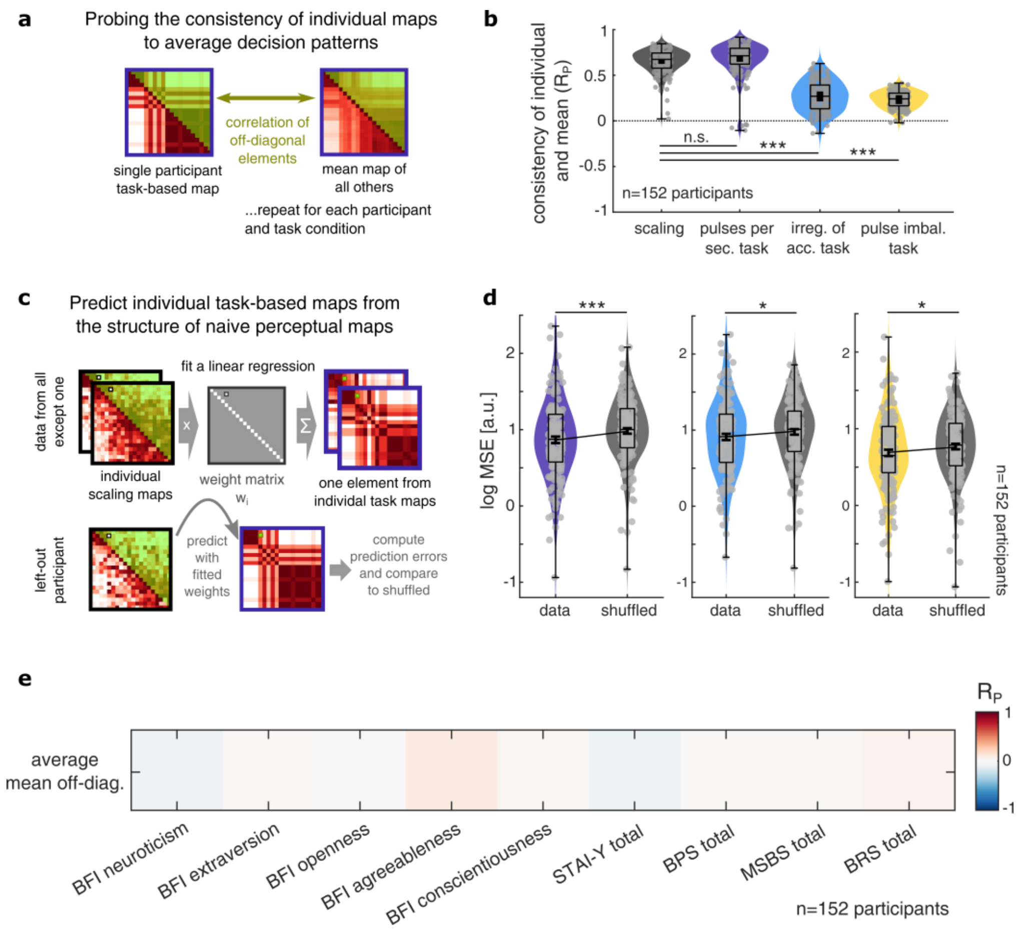

Supplementary Figure 2 – Consistency of individual structures of task-naïve and task-based perceptual maps: (**a**) To test how pronounced perceptual trends were expressed among all participants in the different discrimination tasks, we quantified the consistency of task-based maps by computing correlations between the individual map estimates to the mean map in each condition (only off-diagonal elements were considered (highlighted in green); see Methods). (**b**) Distributions of correlations between individual and mean task-based maps (for all violin/box plots: grey dots represent 152 participants; thin lines indicate the median, the quartiles around the median, and the whiskers indicate top and bottom quartiles; thick black indicates the mean +-SEM; ***: p<0.001 in t-test vs. scaling). The consistency of individual maps to the average map structure was lower for the difficult tasks (*irregularity* and *imbalance*), but higher for the easy task (*pulses per second*), that was comparable to the consistency of the task-naïve condition (mean consistency (R_P) ±SD of n=152 participants: scaling 0.650±0.135, pulses 0.677±0.185, irregularity 0.268±0.163, imbalance 0.231±0.094; t-test vs. scaling: pulses p=0.091, imbalance p<0.001, imbalance p<0.001). (**c**) To test if individual task-based maps are predicted by the structures of naïve perceptual maps, we conducted a linear regression predicting the individual task-based similarity estimates from the naïve perceptual map (see Methods). (**d**) Comparing the mean squared error (MSE) of

a regression on the observed vs. shuffled data revealed a significant predictability of task-based perceptual maps (n=152 participants; mean±SD of log-transformed mean squared regression error: pulses observed 0.867±0.519 vs. pulses shuffled 0.983±0.445, irregularity observed 0.910±0.500vs. irregularity shuffled 0. 977±0.445, imbalance observed 0.686±0.511 vs. imbalance shuffled 0.768±0.443; left-tailed t-test of log-transformed mean squared regression error observed vs. shuffled: pulses p<0.001, irregularity: p=0.028, imbalance p=0.010). This indicates that naïve perceptual maps, estimated from mere similarity ratings, can significantly predict the structure of task-based perceptual maps, derived from specific perceptual decisions under varying reinforcement contingencies. (**e**) Correlations between the average off-diagonal similarity, computed as the mean over the perceptual map estimates from all three task conditions, and different psychometric questionnaires. We did not find a significant association of task-based map structure and psychometric features (n=152 participants, all Pearson correlation coefficients with p>0.05). This notion is in line with the absence of significant correlations between psychometric features and the structure of naïve perceptual maps (Seiler et al., 2025, Perceptual and semantic maps in individual humans share structural features that predict creative abilities, *Communications Psychology*).

Supplementary Figure 3

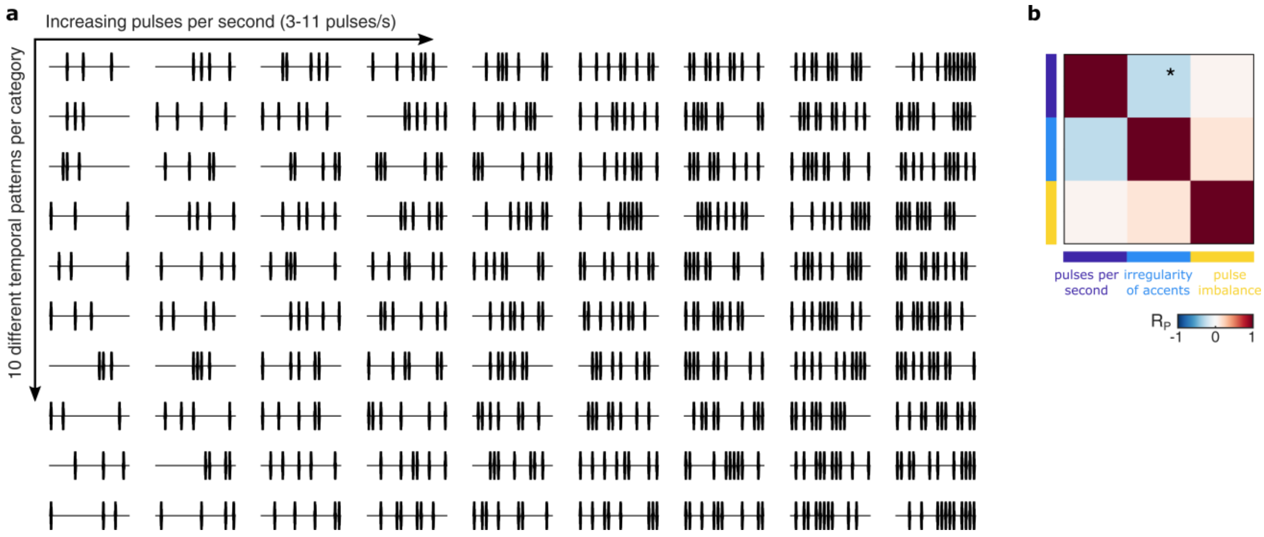

Supplementary Figure 3 – A broad set of pulsed sound stimuli to test perceptual discrimination in mice: (**a**) An extended set of 90 pulsed sound stimuli was used in the auditory discrimination task with mice (also comprising the 20 stimuli used in our human experiments). The pulsed sounds systematically varied in their number of pulses per second (3-11 pulses/s), where for each pulse category, we created ten stimuli with varying temporal patterns. Due to a technical bug in the randomization of sound presentations, only 85 of the 90 pulsed sounds were actually presented to the mice. (**b**) Correlations between the three sound features – *pulses per second*, *irregularity of accents* and *pulse imbalance* – across all 90 sounds. Only *pulses per second* and *irregularity of accents* show a weak negative correlation (R_P=-0.264, p=0.012), while the other features do not exhibit a significant correlation (*pulses per second* vs. *pulse imbalance*: R_P=0.058, p=0.589, *irregularity of accents* vs. *pulse imbalance*: R_P=0.146, p=0.169).

Supplementary Figure 4

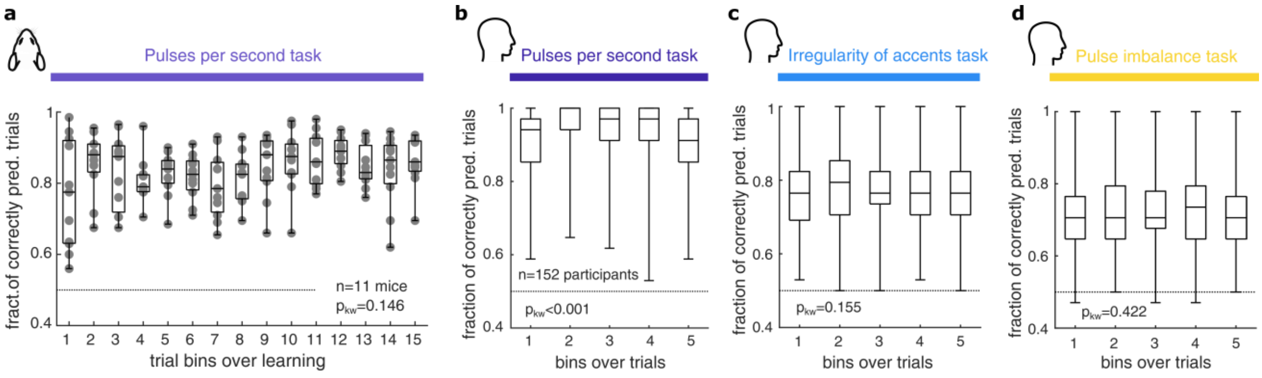

Supplementary Figure 4 – Stable goodness of logistic regression models to assess decision factors over the course of learning: (**a**) Stable goodness of the regression model over all the trial bins across learning (box plots: grey dots represent n=11 single mice; lines indicate the median, the quartiles around the median, and the whiskers indicate top and bottom quartiles; p-value refers to a Kruskal-Wallis test). (**b-d**) Equivalent analyses for the regression goodness in the human tasks (n=152 participants, reported p-values refer to Kruskal-Wallis tests). For the regression in the *pulses per second* task, we observed a dynamic with a slightly higher regression goodness in the middle of the task (trial bins 2-4).

Supplementary Tables

Supplementary Table 1

	**Healthy student cohort** (n = 152)
**Gender**	
Male	32 (21.1%)
Female	120 (78.9%)
**Age** (years)	
Mean	22.8
Standard deviation	4.9
**BMI**	
Mean	23.7
Standard deviation	4.5
**Active neuro psychiatric disorder**	
No	152 (100%)
**Handedness**	
Right	152 (100%)
Left	0 (0%)

Supplementary Table 1 – Demographic characteristics of the study cohort
