# Supplementary material for: Auditory perceptual maps in humans and mice share common structures and predict perceptual decisions in discrimination learning
Source: Commun Psychol. 2026 Jun 20;4:98. doi: 10.1038/s44271-026-00485-w (PMC13282483; doi:10.1038/s44271-026-00485-w)
Supplement: Supplementary file 3 — Reporting Summary [file 44271_2026_485_MOESM3_ESM.pdf]

nature portfolio

Corresponding author(s): Johannes P.-H. Seiler, Simon Rumpel

Last updated by author(s): May 19, 2026

# Reporting Summary

Nature Portfolio wishes to improve the reproducibility of the work that we publish. This form provides structure for consistency and transparency in reporting. For further information on Nature Portfolio policies, see our Editorial Policies and the Editorial Policy Checklist.

## Statistics

For all statistical analyses, confirm that the following items are present in the figure legend, table legend, main text, or Methods section.

| n/a | Confirmed | |
|---|---|---|
| ☐ | ☒ | The exact sample size (*n*) for each experimental group/condition, given as a discrete number and unit of measurement |
| ☐ | ☒ | A statement on whether measurements were taken from distinct samples or whether the same sample was measured repeatedly |
| ☐ | ☒ | The statistical test(s) used AND whether they are one- or two-sided<br>*Only common tests should be described solely by name; describe more complex techniques in the Methods section.* |
| ☐ | ☒ | A description of all covariates tested |
| ☐ | ☒ | A description of any assumptions or corrections, such as tests of normality and adjustment for multiple comparisons |
| ☐ | ☒ | A full description of the statistical parameters including central tendency (e.g. means) or other basic estimates (e.g. regression coefficient) AND variation (e.g. standard deviation) or associated estimates of uncertainty (e.g. confidence intervals) |
| ☐ | ☒ | For null hypothesis testing, the test statistic (e.g. *F*, *t*, *r*) with confidence intervals, effect sizes, degrees of freedom and *P* value noted<br>*Give P values as exact values whenever suitable.* |
| ☒ | ☐ | For Bayesian analysis, information on the choice of priors and Markov chain Monte Carlo settings |
| ☐ | ☒ | For hierarchical and complex designs, identification of the appropriate level for tests and full reporting of outcomes |
| ☐ | ☒ | Estimates of effect sizes (e.g. Cohen's *d*, Pearson's *r*), indicating how they were calculated |

*Our web collection on statistics for biologists contains articles on many of the points above.*

## Software and code

Policy information about availability of computer code

| | |
|---|---|
| Data collection | Custom written MATLAB program (The Mathworks Inc., Natick, Massachusetts, USA, version R2022a). |
| Data analysis | All analyses were conducted using the MATLAB® statistics and machine learning toolbox (The Mathworks Inc., Natick, Massachusetts, USA, version R2022a) |

For manuscripts utilizing custom algorithms or software that are central to the research but not yet described in published literature, software must be made available to editors and reviewers. We strongly encourage code deposition in a community repository (e.g. GitHub). See the Nature Portfolio guidelines for submitting code & software for further information.

## Data

Policy information about availability of data

All manuscripts must include a data availability statement. This statement should provide the following information, where applicable:

- Accession codes, unique identifiers, or web links for publicly available datasets
- A description of any restrictions on data availability
- For clinical datasets or third party data, please ensure that the statement adheres to our policy

*Provide your data availability statement here.*

## Research involving human participants, their data, or biological material

Policy information about studies with human participants or human data. See also policy information about sex, gender (identity/presentation), and sexual orientation and race, ethnicity and racism.

| | |
|---|---|
| Reporting on sex and gender | We used self-report assessments, asking subjects for their gender. We did not specifically assess potential discrepancies between sex and gender. We neither conducted any gender-specific analysis, nor tested for gender differences. Our sample comprised 32 subjects reporting male gender and 120 subjects reporting female gender. |
| Reporting on race, ethnicity, or other socially relevant groupings | We recruited university students irrespective of their ethnical background. We did not collect any reports on race, ethnicity or other social factors. |
| Population characteristics | Apart from the gender distributions, described above, we assessed the age of all participants in our study: mean: 22.8 years, SD: 4.9 years. |
| Recruitment | Healthy students from the University of Mainz were recruited via an online recruiting system to participate in our study (Greiner, 2015, Subject pool recruitment procedures: organizing experiments with ORSEE, Journal of the Economic Science Association). The recruiting system was used to inform subjects about the study and ask them to voluntarily register to participate. |
| Ethics oversight | The study was registered and approved by the local ethics commitee (Ethikkommission der Landesärztekammer Rheinland-Pfalz, processing number 2024-17477). |

Note that full information on the approval of the study protocol must also be provided in the manuscript.

# Field-specific reporting

Please select the one below that is the best fit for your research. If you are not sure, read the appropriate sections before making your selection.

☒ Life sciences ☐ Behavioural & social sciences ☐ Ecological, evolutionary & environmental sciences

For a reference copy of the document with all sections, see nature.com/documents/nr-reporting-summary-flat.pdf

# Life sciences study design

All studies must disclose on these points even when the disclosure is negative.

| | |
|---|---|
| Sample size | The sample size for the human cohort was estimated based on effect sizes, observed in previous experiments, similar to this study. |
| Data exclusions | A total number of 162 healthy students from the University of Mainz was recruited. From the initial sample, 10 participants encountered technical problems during the experiment, leading to lacking or incomplete data and exclusion from our analyses. Thus, a final sample of 152 subjects was used for our analyses. |
| Replication | So far, no replication experiment was conducted. |
| Randomization | The order of presenting the stimuli and trial types in the choice tasks was randomized. Apart from this, this point is not applicable to our study. |
| Blinding | Blinding was not relevant in our study. |

# Reporting for specific materials, systems and methods

We require information from authors about some types of materials, experimental systems and methods used in many studies. Here, indicate whether each material, system or method listed is relevant to your study. If you are not sure if a list item applies to your research, read the appropriate section before selecting a response.

## Materials & experimental systems

| n/a | Involved in the study |
|---|---|
| ☒ | ☐ Antibodies |
| ☒ | ☐ Eukaryotic cell lines |
| ☒ | ☐ Palaeontology and archaeology |
| ☐ | ☒ Animals and other organisms |
| ☒ | ☐ Clinical data |
| ☒ | ☐ Dual use research of concern |
| ☒ | ☐ Plants |

## Methods

| n/a | Involved in the study |
|---|---|
| ☒ | ☐ ChIP-seq |
| ☒ | ☐ Flow cytometry |
| ☒ | ☐ MRI-based neuroimaging |

# Animals and other research organisms

Policy information about studies involving animals; ARRIVE guidelines recommended for reporting animal research, and Sex and Gender in Research

| | |
|---|---|
| Laboratory animals | We obtained an initial sample of 12 wildtype C57BL/6J mice with an age of approximately 6 weeks. In our analyses, we only include data from 11 mice, since one mouse did not learn the discrimination task. |
| Wild animals | n/a |
| Reporting on sex | We only used male mice in our study. |
| Field-collected samples | Mice were kept in groups of four, and housed in 530 scm cages on a 12h light/dark cycle with unlimited access to dry food. Experiments were carried out during the light period. |
| Ethics oversight | All animal experiments were performed in accordance with the German laboratory animal law guidelines for animal research and had been approved by the Landesuntersuchungsamt Rheinland Pfalz (Approval # 23 177-07/G 17-1-051). |

Note that full information on the approval of the study protocol must also be provided in the manuscript.

# Plants

| | |
|---|---|
| Seed stocks | n/a |
| Novel plant genotypes | n/a |
| Authentication | n/a |
