# Supplementary material for: Auditory perceptual maps in humans and mice share common structures and predict perceptual decisions in discrimination learning
Source: Commun Psychol. 2026 Jun 20;4:98. doi: 10.1038/s44271-026-00485-w (PMC13282483; doi:10.1038/s44271-026-00485-w)
Supplement: Supplementary file 1 — Transparent Peer Review file [file 44271_2026_485_MOESM1_ESM.pdf]

nature portfolio

Peer Review File

Auditory perceptual maps in humans and mice share common structures and predict perceptual decisions in discrimination learning

Corresponding Author: Professor Simon Rumpel

This file contains all editorial decision letters in order by version, followed by all author rebuttals in order by version.

Version 0:

Decision Letter:

** Please ensure you delete the link to your author homepage in this e-mail if you wish to forward it to your coauthors **

Dear Professor Rumpel,

Thank you for your patience during the peer-review process. Your manuscript titled "Conserved perceptual maps in humans and mice guide perceptual decisions in discrimination learning" has now been seen by 2 reviewers, and I include their comments at the end of this message. They find your work of interest but raised some important points. We are interested in the possibility of publishing your study in Communications Psychology, but would like to consider your responses to these concerns and assess a revised manuscript before we make a final decision on publication.

We therefore invite you to revise and resubmit your manuscript, along with a point-by-point response to the reviewers. Please highlight all changes in the manuscript text file.

Editorially, we consider is necessary that you address the reviewers' methodological and conceptual concerns. Moreover, the statistical analysis and how it is reported will need to be revised to satisfy the journal's requirements. Examples are included in the attached document. You can find more information here:https://www.nature.com/commspsychol/submit/submission-guidelines#statistical-guidelines.

As you revise the manuscript in response to these issues, please also implement all requests in the attached Mandatory Revision Requests document. All requirements listed in this document need to be fully met, or the work will be returned to you for further revisions without peer review. This workflow is in place to increase the likelihood that the paper will be accepted for publication. It reduces the number of rounds of revision (and review) and ensures that the reviewers vet a version of the article that is compliant with journal policies. If you have any questions regarding the required revisions, please contact the journal prior to resubmission to avoid a negative outcome.

We are committed to providing a fair and constructive peer-review process. Please don't hesitate to contact us if you wish to discuss the revision in more detail.

Please submit the following items:

- Revised manuscript
- Point-by-point response to the referees’ comments
- Mandatory Revision Requests Table (attached).
- Cover letter (as a separate document)

via this link: Link Redacted .

** This url links to your confidential home page and associated information about manuscripts you may have submitted or are reviewing for us. If you wish to forward this email to co-authors, please delete the link to your homepage first **

We hope to receive your revised paper within 8 weeks; please let us know if you aren't able to submit it within this time so that we can discuss how best to proceed. If we don't hear from you, and the revision process takes significantly longer, we may close your file. In this event, we will still be happy to reconsider your paper at a later date, provided it still presents a significant contribution to the literature at that stage.

We look forward to seeing the revised manuscript and thank you for the opportunity to review your work.

Best regards,

Marike

Marike Schiffer, PhD
Chief Editor
Communications Psychology

REVIEWER EXPERTISE:
Reviewer #1 & #2: auditory perception, psychophysics, human & rodent models

REVIEWER REPORTS:

Reviewer #1 (Remarks to the Author):

This manuscript investigates the relationship between task-naïve perceptual similarity maps and reinforcement-based discrimination learning for complex auditory stimuli in humans and mice. By parametrizing pulsed sounds along three dimensions (pulses per second, irregularity of accents, and pulse imbalance), the authors construct perceptual similarity matrices and test whether these naïve perceptual structures predict discrimination performance. A cross-species comparison is presented as evidence for conserved perceptual organization.

The study addresses an interesting question: whether perceptual similarity structures derived under naïve conditions constrain and predict task-based learning, and whether such structures are conserved across species. The attempt to bridge psychophysical similarity judgments with operant discrimination paradigms, and to extend this framework to rodents, is conceptually appealing. However, several conceptual, methodological, analytical, and interpretative issues limit the strength of the conclusions. In its current form, the manuscript requires substantial revision before it can be considered for publication.

Mario Treviño

Title and Abstract

The title is relatively generic and does not clearly reflect the auditory specificity of the study, nor its main empirical findings (e.g., the dominance of pulse rate and the feature-specific nature of predictive relationships).
The abstract overstates the extent of cross-species conservation. Humans were tested on three acoustic dimensions, whereas mice were assessed only on pulses per second. Therefore, claims of “similar perceptual maps” or evolutionary preservation should be tempered. It would be important to explicitly report sample sizes in the abstract (n = 152 humans; n = 11 mice), as these are central for evaluating generalizability (one possibility is to remove the entire mouse section, or increase the sample size).
Several statements emphasize “stable perceptual architecture” in a somewhat repetitive manner. These claims should be aligned more closely with the actual strength of the evidence.

Conceptual Framing: What Is a “Perceptual Map”?

A central concern is conceptual clarity. The term “perceptual map” is used extensively, yet remains insufficiently defined at the outset. It is unclear whether this term refers to:

- an abstract psychophysical construct derived from similarity judgments,
- a behavioral similarity matrix,
- or a putative neural representational space (e.g., tonotopic or network-level coding).

The introduction discusses representational maps in a broad sense, referencing cognitive science traditions, but does not clearly explain how the present study operationalizes and measures such maps. Only upon reaching Figure 1a does it become clear that the “perceptual map” corresponds to a mean similarity matrix derived from pairwise judgments.

If the map is purely behavioral and derived from similarity ratings, this should be stated explicitly and early. If no direct neural measurements are involved, the terminology should avoid implying cytoarchitectonic or physiological mappings unless such links are explicitly hypothesized. The use of additional terms such as "structure," "architecture," or "shared structural variability" further increases ambiguity. These terms should be standardized and clearly defined. If "perceptual similarity matrix" is the most accurate descriptor, adopting this terminology consistently may improve clarity.
Relatedly, the distinction between "task-naïve" and "task-based" conditions requires clearer definition. How did participants respond in each condition? What were the instructions? How many trials were used? After extensive exposure during similarity judgments, in what sense do participants remain "naïve"? These methodological details are essential to interpret the construct.

Theoretical Positioning and Evolutionary Claims

The introduction presents perceptual maps as general representational frameworks but does not sufficiently develop the evolutionary rationale underlying the cross-species comparison. If conservation across humans and mice is a central claim, the manuscript would benefit from engagement with comparative auditory literature (e.g., work on mammalian auditory evolution by Manley or Fritzsch). Currently, the evolutionary argument appears underdeveloped relative to the strength of the conclusions.
Moreover, stating that perceptual maps are "preserved across species" suggests identity. The evidence instead supports partial, feature-specific functional similarity under specific task conditions. It would be more precise to refer to functional congruence or partial conservation rather than preservation of underlying maps.

Methodological Issues
Auditory screening in humans.

It is not stated whether participants' auditory capacity was screened (e.g., hearing thresholds, self-report of impairment). In visual psychophysics, acuity is routinely controlled. Without such screening, individual differences in hearing could bias similarity judgments or discrimination performance.

Sample sizes.

The disparity between human ($n \approx 150$) and mouse ($n = 11$) samples requires justification. While animal studies often have smaller samples, the strength of cross-species claims should be framed accordingly.

Stimulus feature selection.

The choice of three acoustic features (pulses, irregularity, imbalance) appears somewhat arbitrary. A stronger justification is needed for selecting these dimensions over other auditory features (e.g., spectral content, amplitude modulation, envelope characteristics), particularly given the extensive literature on auditory cortical coding.

Reinforcement rules.

The rationale for the three reinforcement rules should be clearly articulated. Why were these specific task structures chosen, and how do they test the theoretical claims?
Ethical considerations.

Water deprivation in mice is common but should be explicitly justified, with clear reporting of welfare monitoring procedures.

Statistical and Analytical Concerns
Clustering and map structure.

Similarity matrix clustering is described descriptively. Statistical validation (e.g., hierarchical clustering metrics, silhouette scores) would strengthen claims about dominant clusters.

Regression modeling.

Linear regression assumes additive, linear contributions of features. Perceptual spaces may exhibit non-linear interactions. Testing interaction terms or non-linear models would provide greater confidence. Multicollinearity diagnostics should also be reported.

Learning curves.

Sigmoid fits are appropriate, but goodness-of-fit metrics (R^2, AIC) should be provided and compared against alternative models (e.g., linear fits).

Correlation strength and multiple comparisons.

Many reported correlations are weak to moderate (e.g., $RP = 0.238$). Effect sizes should be discussed explicitly. Given the number of correlations performed, stricter correction procedures (e.g., Bonferroni) or bootstrapped confidence intervals would provide a more robust assessment. Non-significant correlations, particularly for imbalance, should not be

overinterpreted.

Feature-specific conservation.

The high cross-species correlation for pulses (RP = 0.896) is compelling. However, irregularity (RP = 0.520) and imbalance (RP = 0.222) show markedly weaker alignment. Thus, claims of “shared key structures” should be framed as feature-dependent rather than general.

Species comparisons.

Formal statistical tests comparing human and mouse similarity matrices (e.g., Mantel tests or matrix correlations with permutation tests) would strengthen conservation claims.

Interpretation and Potential Circularity

The finding that pulse rate dominates naïve maps and also yields fastest learning may partly reflect circularity. If the naïve perceptual space is already strongly organized around pulse rate, superior discrimination along this dimension is expected. The manuscript should discuss this possibility and clarify whether the data truly demonstrate predictive power beyond baseline feature salience.

The relationship between “perceptual maps” and “perceptual learning” is also insufficiently clarified. Are maps pre-existing frameworks that constrain learning, or are they plastic and shaped by experience? The manuscript would benefit from situating findings within established theories of perceptual learning.

Cross-Species and Personality Analyses
The link between perceptual similarity weights and personality measures is intriguing but under-theorized. What is the proposed neurobiological mechanism linking similarity structure to creativity or personality traits? Moreover, if conservation across species is emphasized, how would analogous personality constructs be operationalized in rodents?
Species differences in GLM parameters (e.g., initial biases in mice) should be discussed explicitly. Are these methodological (head-fixed vs. free-moving) or biological?

Discussion and Limitations

The Discussion primarily reiterates results rather than synthesizing broader implications. It would benefit from engagement with:

• perceptual learning theory,
• representational geometry approaches,
• and potential neural correlates (e.g., future neuroimaging or calcium imaging studies).

Limitations should be more fully acknowledged, including:

• student-biased human sample,
• potential order effects,
• limited mouse sample size,
• and restriction of cross-species comparison to one acoustic feature.

Reviewer #2 (Remarks to the Author):

This paper studied the perceptual similarity to a set of pulsed sound stimuli using psychophysics measurement under both naive and task contexts, and compared the perception between humans and mice, showing interesting cross-species conservation. The study provides a valuable demonstration and dataset of investigating perceptual changes during learning, computing comparable perceptual map across different behavioral paradigms, and linking different species for their shared cognitive processes.

1. Line 76: Is this perceptual similarity rating stable? What is the reliability of the rating? Measuring perceptual similarity with a scale is more subject to fluctuations and drifts of individual criterion and such, when compared to paradigms like match-to-sample.

2. Line 99-102: The lack of pairwise correlations between stimulus features suggests that the stimulus is approximately orthogonal, but does not imply independent perceptual encoding. If the goal is to assess the feature independency in perceptual encoding, explicit implementing interaction terms in the regression model is needed, which would affect the

interpretation of other results.

3. Line 151: In future studies, it will be interesting to collect verbal description of rules more frequently to see when the correct rule emerges and how it corresponds to the performance improvement.

4. Line 199: Were the initial learning trials under tasks excluded when calculating the difference in response probabilities? Same question for mouse data. What’s the criterion if any?

5. The reported similarity of perceptual maps across contexts (naive vs task) and across species is interesting. However, with the current experiment, it is difficult to disentangle whether the observed similarity is driven by the underlying structure of the stimulus features, or by the internal perceptual maps. The map similarity/predictability is mainly quantified with correlation, while a high correlation score here might be a reflection of the shared stimulus features, or be dominated by one of the features (pulse number). It would be helpful to more explicitly address the contribution of stimulus features in the analysis, as well as if they have tested generalization, such as by predicting the response to an independent stimulus set.

6. Line 358: Could the authors please elaborate on why the results support speed-accuracy tradeoffs (SAT)? SAT is a decision strategy, either innate or task-induced, where speed and accuracy are inversely related. Here I don’t observe such a tradeoff.

7. Line 369-372: To me, the findings in this paper seem to be equally consistent with both scenarios. The correlation between naive and task-based maps could also arise from the constraints on how easily perceptual maps can be transformed during learning. The boundary between ‘representational map formation’ and ‘readout process’ is difficult to draw based on behavioral data alone, as the two are relative concepts that are functionally and even neurally intertwined. Depending on the hierarchical level of the stimulus representation, the timescale of ‘remodeling’ may be fast or slow, making the claim in line 375-377 less compelling as evidence against representational change. It’s good that the authors made it explicit that due to the lack of neural data, they cannot fully distinguish the two scenarios mentioned in line 363-367.

8. The participants were told there were 3 task rules but NOT told what the 3 rules were, correct? Could mention it early on in the method.

9. I understand this paper focused on learning, but it would be interesting in future studies to test humans with explicit instructions on what the task rules are and make comparisons. Some category-learning processes, such as medical image learning or chord learning, usually require prolonged training and explicit guidance. This situation might be more comparable to the pulse imbalance condition, and could be quantatively or even qualitatively different from learning to discriminate features that are already highly discernable from naive perceptual space.

10. Line 694-701: In the quantification of the explicit understanding, have the authors considered/controlled in anyway for the difficulty of task rule description? I would imagine describing the pulse number rule is quite easy while the other two is tricky to articulate. Especially the example reports for the irregularity of accents and pulse imbalance appear vague and potentially have conceptual/expressive overlap across rules. This poses challenges in interpreting the results in line 157.

* TRANSPARENT PEER REVIEW: Communications Psychology uses a transparent peer review system. This means that we publish the editorial decision letters including Reviewers' comments to the authors and the author rebuttal letters online as a supplementary peer review file. However, on author request, confidential information and data can be removed from the published reviewer reports and rebuttal letters prior to publication. If your manuscript has been previously reviewed at another journal, those Reviewers' comments would not form part of the published peer review file.

** Visit Nature Research's author and referees' website at www.nature.com/authors for information about policies, services and author benefits**

Communications Psychology is committed to improving transparency in authorship. As part of our efforts in this direction, we are now requesting that all authors identified as ‘corresponding author’ create and link their Open Researcher and Contributor Identifier (ORCID) with their account on the Manuscript Tracking System prior to acceptance. ORCID helps the scientific community achieve unambiguous attribution of all scholarly contributions. You can create and link your ORCID from the home page of the Manuscript Tracking System by clicking on ‘Modify my Springer Nature account’ and following the instructions in the link below. Please also inform all co-authors that they can add their ORCIDs to their accounts and that they must do so prior to acceptance.
https://www.springernature.com/gp/researchers/orcid/orcid-for-nature-research

For more information please visit http://www.springernature.com/orcid

If you experience problems in linking your ORCID, please contact the Platform Support Helpdesk.

Version 1:

Decision Letter:

** Please ensure you delete the link to your author homepage in this e-mail if you wish to forward it to your coauthors **

Dear Professor Rumpel,

Your manuscript titled "Auditory perceptual maps in humans and mice share common structures and predict perceptual decisions in discrimination learning" has now been seen by our reviewers, whose comments appear below. In light of their advice I am delighted to say that we are happy, in principle, to publish a suitably revised version in Communications Psychology.

We therefore invite you to revise your paper one last time to address the remaining concerns of our reviewers and a list of editorial requests. At the same time we ask that you edit your manuscript to comply with our format requirements and to maximise the accessibility and therefore the impact of your work.

EDITORIAL REQUESTS:

Please review our specific editorial comments and requests regarding your manuscript in the attached "Editorial Requests Table". Please outline your response to each request in the right hand column. Please upload the completed table with your manuscript files as a Related Manuscript file.

If you have any questions or concerns about any of our requests, please do not hesitate to contact me.

SUBMISSION INFORMATION:

In order to accept your paper, we require the files listed here https://www.nature.com/documents/commsj-file-checklist.pdf .

OPEN ACCESS:

Communications Psychology is a fully open access journal. Articles are made freely accessible on publication. For further information about article processing charges, open access funding, and advice and support from Nature Research, please visit https://www.nature.com/commspsychol/open-access

At acceptance, you will be provided with instructions for completing the open access licence agreement on behalf of all authors. This grants us the necessary permissions to publish your paper. Additionally, you will be asked to declare that all required third party permissions have been obtained, and to provide billing information in order to pay the article-processing charge (APC).

* TRANSPARENT PEER REVIEW: Communications Psychology uses a transparent peer review system. On author request, confidential information and data can be removed from the published reviewer reports and rebuttal letters prior to publication. If you are concerned about the release of confidential data, please let us know specifically what information you would like to have removed. Please note that we cannot incorporate redactions for any other reasons.

Please use the following link to submit the above items:
Link Redacted
** This url links to your confidential home page and associated information about manuscripts you may have submitted or be reviewing for us. If you wish to forward this email to co-authors, please delete the link to your homepage first **

We hope to hear from you within two weeks; please let us know if you need more time.

Best regards,

Marike

Marike Schiffer, PhD
Chief Editor
Communications Psychology

REVIEWERS' COMMENTS:

Reviewer #1 (Remarks to the Author):

Minor revisions. The manuscript is substantially improved. The authors addressed the conceptual ambiguity around "perceptual map," added proper statistical validation of the clustering, compared sigmoid fits to linear alternatives, and expanded the Discussion to engage with perceptual learning theory. Two small issues remain: the self-report hearing screening should be acknowledged as a limitation of the psychophysical validity of the data, and the asymmetry in trial exclusion across species should be noted explicitly. Neither requires new data or reanalysis. Both can be resolved with one or two sentences in the Limitations section. Congratulations on your nice paper!

Mario Treviño

Reviewer #2 (Remarks to the Author):

I have no further comments. The authors have addressed my concerns by including more information, adjusting the wording, and explicitly incorporating some of the limitations of their study.

** Visit Nature Research's author and referees' website at www.nature.com/authors for information about policies, services and author benefits**

Open Access This Peer Review File is licensed under a Creative Commons Attribution 4.0 International License, which permits use, sharing, adaptation, distribution and reproduction in any medium or format, as long as you give appropriate credit to the original author(s) and the source, provide a link to the Creative Commons license, and indicate if changes were made.
In cases where reviewers are anonymous, credit should be given to 'Anonymous Referee' and the source.
The images or other third party material in this Peer Review File are included in the article’s Creative Commons license, unless indicated otherwise in a credit line to the material. If material is not included in the article’s Creative Commons license and your intended use is not permitted by statutory regulation or exceeds the permitted use, you will need to obtain permission directly from the copyright holder.
To view a copy of this license, visit https://creativecommons.org/licenses/by/4.0/

Rebuttal Letter: Manuscript "Auditory perceptual maps in humans and mice share common structures and predict perceptual decisions in discrimination learning" – Review 1

With this, we would like to re-submit our manuscript entitled "Auditory perceptual maps in humans and mice share common structures and predict perceptual decisions in discrimination learning" (previous title: "Conserved perceptual maps in humans and mice guide perceptual decisions in discrimination learning"; ID COMMSPSYCHOL-26-0085-T) for publication as a research article in *Communications Psychology*.

We thank the reviewers for their insightful comments and constructive criticism. We believe that the reviews and the subsequent adjustments have significantly improved the quality of our manuscript.
The respective revisions are marked by blue font color in the attached main text and in this rebuttal letter.

In the following, we address the reviews point by point:

> ***Reviewer #1:***
>
> *This manuscript investigates the relationship between task-naïve perceptual similarity maps and reinforcement-based discrimination learning for complex auditory stimuli in humans and mice. By parametrizing pulsed sounds along three dimensions (pulses per second, irregularity of accents, and pulse imbalance), the authors construct perceptual similarity matrices and test whether these naïve perceptual structures predict discrimination performance. A cross-species comparison is presented as evidence for conserved perceptual organization.*
> *The study addresses an interesting question: whether perceptual similarity structures derived under naïve conditions constrain and predict task-based learning, and whether such structures are conserved across species. The attempt to bridge psychophysical similarity judgments with operant discrimination paradigms, and to extend this framework to rodents, is conceptually appealing. However, several conceptual, methodological, analytical, and interpretative issues limit the strength of the conclusions. In its current form, the manuscript requires substantial revision before it can be considered for publication.*

Thank you.

> *Title and Abstract*
> *The title is relatively generic and does not clearly reflect the auditory specificity of the study, nor its main empirical findings (e.g., the dominance of pulse rate and the feature-specific nature of predictive relationships).*

We appreciate this comment, showing us that we should formulate our title more specifically. To address the comment, we have revised our title according to the reviewer's suggestions: "Auditory perceptual maps in humans and mice share common structures and predict perceptual decisions in discrimination learning".

> *The abstract overstates the extent of cross-species conservation. Humans were tested on three acoustic dimensions, whereas mice were assessed only on pulses per second. Therefore, claims of "similar perceptual maps" or evolutionary preservation should be tempered. It would be important to explicitly report sample sizes in the abstract (n = 152 humans; n = 11 mice), as these are central for evaluating generalizability (one possibility is to remove the entire mouse section, or increase the sample size).*

We agree with the reviewer that we should report the differences in size between the human and mouse sample, and the different types of assessing perceptual maps in the abstract of our manuscript, in order to clarify how cross-species comparisons were conducted. We therefore revised our abstract, adding this information and specifying that mouse perceptual maps were solely assessed in a task-based setting, whereas human perceptual maps were assessed in both, task-naïve and task-based, settings (l. 19 ff.):

"Here, we assess task-naïve perceptual maps from similarity judgments of pulsed sound stimuli, and compare them to perceptual maps obtained from multiple discrimination tasks in humans (n = 152) and mice (n = 11). We find that task-naïve maps predict how well humans discriminate sounds, how quickly they learn, and which stimulus features guide their choices. Moreover, naïve and task-based maps share key structures, suggesting a stable perceptual map architecture throughout learning. Remarkably, human task-based and task-naïve perceptual maps share key features and structures with the task-based perceptual maps of mice, indicating congruent structures of auditory perception across species. Together, our results indicate that perception relies on robust internal maps that provide a common framework to flexibly guide behavior in changing environments."

> *Several statements emphasize "stable perceptual architecture" in a somewhat repetitive manner. These claims should be aligned more closely with the actual strength of the evidence.*

In our study, we demonstrate a significant degree of similarity between perceptual maps assessed in a task-naïve and a task-based setting. Morerover, we find that perceptual decision strategies adapt over the course of learning, suggesting that while perceptual relations are largely maintained, individuals adjust their readout of these maps during learning. This notion is in line with previous studies, showing a high degree of stability in representational maps under basal and conditions[1] as well as during learning[2-5]. Together, this line of evidence has led to our statement about "stable perceptual architectures", which we deem adequate to describe our observations.
We nevertheless took the reviewer's comment as a motive to enhance the clarity of our wording. We have therefore changed the wording in our abstract to "…suggesting a stable perceptual map architecture throughout learning." (l. 23 f.), and further replaced the term "stable perceptual architecture" with "stable structure" at later parts of the manuscript.

> *Conceptual Framing: What Is a "Perceptual Map"?*
> *A central concern is conceptual clarity. The term "perceptual map" is used extensively, yet remains insufficiently defined at the outset. It is unclear whether this term refers to:*
> *• an abstract psychophysical construct derived from similarity judgments,*
> *• a behavioral similarity matrix,*
> *• or a putative neural representational space (e.g., tonotopic or network-level coding).*
>
> *The introduction discusses representational maps in a broad sense, referencing cognitive science traditions, but does not clearly explain how the present study operationalizes and measures such maps. Only upon reaching Figure 1a does it become clear that the "perceptual map" corresponds to a mean similarity matrix derived from pairwise judgments.*
> *If the map is purely behavioral and derived from similarity ratings, this should be stated explicitly and early. If no direct neural measurements are involved, the terminology should avoid implying cytoarchitectonic or physiological mappings unless such links are explicitly hypothesized. The use of*

additional terms such as “structure,” “architecture,” or “shared structural variability” further increases ambiguity. These terms should be standardized and clearly defined. If “perceptual similarity matrix” is the most accurate descriptor, adopting this terminology consistently may improve clarity.

Thank you for this comment, making us clear that we should better explain our definition of a perceptual map.
In short: We use the term representational map as an abstract cognitive concept how representational tokens (including representations of sensory stimuli) are organized at various hierarchical levels in the brain. The structure of such representational maps can be experimentally estimated using psychophysics (as we use it in our study), but interestingly also can be estimated using population recordings of neuronal activity, as they seem to have a correlate encoded at the activity level (not used in this study).
In our study, we use the term *perceptual map* to describe a specific type of *representational map* at the perceptual level that we estimate using psychophysical assessments in the form of similarity matrices. The similarity matrices serve here as our experimentally accessible proxy for the abstract representational map that in principle could have also other forms such as distance matrices or graph structures.

To address the reviewer’s comment, we revised the introduction section of our manuscript to clarify this definition (l. 44 ff.):

“Originally developed in the cognitive sciences, the abstract concept of representational maps describes the organization and encoding of the relatedness between representational tokens in cognitive space at various levels of hierarchy. In experiments, the structure of representational maps has been classically assessed in the psychophysical domain, e.g. based on simple ratings of perceived similarity[6-8]. Over recent years this approach has also found broad support in the neurosciences[9,10]. Specifically, it was shown that perceptual representational maps assessed by self-reports or behavioral similarity estimates, share key structures with neuronal representational maps[3,11-13], assessed by comparing the similarities between stimulus-related patterns of brain activity[14]. In this manuscript, we use the term *perceptual map* to refer to the representational map that encodes perceptual relatedness of the sound stimuli used in this study, experimentally estimated by assessing their perceptual similarities. Specifically, these perceptual maps are estimated from individual representational similarity matrices, comprising all individual pairwise similarity estimates between a defined set of stimuli, adhering to the procedures of previous studies (see ref[10] for a review).
Typically, assessments of perceptual maps are conducted under basal conditions, without the context of a task and without reinforcement. In contrast, studies on perceptual decision-making across species often employ tasks in which reinforcement is crucial[15]. Thus, it remains unclear in how far perceptual maps estimated in different contexts are related. Do naïve and task-based perception diverge, suggesting the implementation of independent perceptual maps for both conditions?“

Relatedly, the distinction between “task-naïve” and “task-based” conditions requires clearer definition. How did participants respond in each condition? What were the instructions? How many trials were used? After extensive exposure during similarity judgments, in what sense do participants remain “naïve”? These methodological details are essential to interpret the construct.

Thank you for pointing this out. Indeed, we termed participants performing a reinforcement-free scaling task as “task naïve”, and considered only those involved in the reinforcement-based discrimination task as

no longer “task naïve”. This can cause confusion, obviously. To address this issue, we replaced the term “scaling task” with “similarity rating”, or “scaling paradigm” throughout our manuscript.

In addition, we expanded the corresponding text sections in the Introduction and Results part of our manuscript, detailing the information on the assessment of perceptual maps under task-naïve and task-based conditions. Specifically, we detail the information on the number of trials and instructions in each task (see e.g. l. 162, 183) and further expand our explanation how task performance is used to operationalize perceptual maps under task-naïve and task-based conditions.

Introduction section (l. 66 ff.):
“We conduct assessments of task-naïve perceptual maps using a reinforcement-free scaling paradigm in humans, asking participants to rate the perceived pairwise similarities for a fixed set of sounds. In addition, we independently assess task-based perceptual maps, using reinforcement learning to categorically discriminate sound stimuli. In these reinforcement-based discrimination tasks participants learned to classify sounds into two categories defined by a specific sound property in order to obtain monetary reward.”

Results section (l. 529 ff., 542 ff.):
“In our study, we sought to investigate and compare the structures of task-naïve and task-based perceptual maps for a fixed set of complex sound stimuli. For this purpose, we recruited a sample of 152 students free from active hearing impairment and neuropsychiatric disorders (demographics detailed in Supplementary Table 1), and devised a paradigm in which participants first underwent a reinforcement-free scaling task with a set of pulsed sound stimuli, and subsequently completed choice tasks to discriminate the same set of stimuli under three different reinforcement rules (Figure 1a; Methods). Note, that we refer in this study to participants that have not yet been involved in the reinforcement-based discrimination task as *task-naïve.*
[…]
First, we investigated perceptual maps in task-naïve conditions free from reinforcement, using the scaling task, in which participants scored all pairwise similarities between a set of 20 different pulsed sounds. Specifically, participants underwent 210 trials, each of which comprised the presentation of a given pair of sounds with the subsequent instruction to rate the degree of similarity between the sampled sounds on a visual analog scale (see Methods for task details). Note that the order of stimuli was randomized to avoid confounding of the perceptual ratings by sequence biases. These pairwise similarity ratings allowed us to construct a 20-by-20 similarity matrix for each participant which we further used as a proxy of an individual’s perceptual map (Methods).”

In addition, we adjusted the wording and added information at several points of our Methods section to make our operationalization clearer (e.g. l. 153, 170).

> *Theoretical Positioning and Evolutionary Claims*
> *The introduction presents perceptual maps as general representational frameworks but does not sufficiently develop the evolutionary rationale underlying the cross-species comparison. If conservation across humans and mice is a central claim, the manuscript would benefit from engagement with comparative auditory literature (e.g., work on mammalian auditory evolution by Manley or Fritzsch). Currently, the evolutionary argument appears underdeveloped relative to the strength of the conclusions.*

Thank you for this note. Representational maps constitute a general framework to quantitatively assess the perceptual and/or neuronal relations between different stimuli. With this general nature, representational maps constitute a promising approach to compare representational structures – and with this also sensory encoding – between individual, but also between different species. The translational potential of the representational map approach has for instance been demonstrated by Kriegeskorte and colleagues, systematically comparing the visual representations in monkey and man, finding shared key structures across species[14].
Nevertheless, we agree with the reviewer that we should mention this aspect more explicitly. We have therefore added a phrase to the Introduction section, mentioning this aspect. In addition, we have added a reference to the work of Manley and Fritzsch as suggested (l. 38 ff.):

"Besides their implications on individual behavior, representational maps serve as an instrumental approach to compare encoded sensory information between different species. For instance, a previous study comparing the representational map structures in humans and monkeys identified shared visual representations of animate or inanimate objects[14]. Similar cross-species comparison of representational maps appears promising for the auditory system, in which a high degree of functional convergence and gene homology has been described[16-18].
"

> *Moreover, stating that perceptual maps are "preserved across species" suggests identity. The evidence instead supports partial, feature-specific functional similarity under specific task conditions. It would be more precise to refer to functional congruence or partial conservation rather than preservation of underlying maps.*

To address this comment, we toned down the wording in our manuscript, changing the phrase "conserved perceptual maps" to "similar perceptual maps", or to "congruent perceptual maps" as suggested (e.g. see l. 25, 722 f.).

> *Methodological Issues*
> *Auditory screening in humans.*
> *It is not stated whether participants' auditory capacity was screened (e.g., hearing thresholds, self-report of impairment). In visual psychophysics, acuity is routinely controlled. Without such screening, individual differences in hearing could bias similarity judgments or discrimination performance.*

Thank you for raising this important aspect. Indeed, we verified that all participants had regular hearing abilities during the recruitment procedure. We used self-reports to verify that all included participants had normal (or corrected) hearing ability.
We added a note on this point to the Results and Methods section:

Results section (l. 530 f.):
"For this purpose, we recruited a sample of 152 students free from active hearing impairment and neuropsychiatric disorders."

Methods section (l. 122 ff.):

“Exclusion criteria were the presence of impaired hearing ability, active psychiatric or neurological disorders, or insufficient German language skills, all assessed through self-reports.”

Sample sizes.
The disparity between human (n ≈ 150) and mouse (n = 11) samples requires justification. While animal studies often have smaller samples, the strength of cross-species claims should be framed accordingly.

In general, our experiments with mice took several weeks until they were reached a sufficient discrimination performance for one task contingency. In contrast, humans learned three different contingencies on a single day of experiments. Thus, the extensive time effort to train mice in a perceptual discrimination task limits the sample size, as true for many behavioral studies with mice. We agree with the reviewer that this point should be mentioned explicitly in the discussion of our study. Therefore, we added this aspect to the Limitations section in our discussion (l. 938 ff.):

“In addition, compared to our human sample undergoing perceptual discrimination tasks for three different stimulus features, our mouse cohort was substantially smaller and only underwent perceptual discrimination of one single stimulus feature. Replication of our experiments, using a larger mouse sample and more extensive discrimination tasks of more stimulus features could in the future corroborate the effects we observed in our cross-species comparisons.“

Stimulus feature selection.
The choice of three acoustic features (pulses, irregularity, imbalance) appears somewhat arbitrary. A stronger justification is needed for selecting these dimensions over other auditory features (e.g., spectral content, amplitude modulation, envelope characteristics), particularly given the extensive literature on auditory cortical coding.

Thank you for this comment, showing us that we should make our rationale to choose the stimulus features clearer. In a recent study[19], we quantified pulsed sound stimuli by a rich and diverse set of different features, and tested to what degree each feature was represented at the level of the auditory cortex. In this study, we identified the features pulse number, irregularity and temporal imbalance as being dominantly represented, observing that the neural representation of each of these features allowed to predict stimulus-specific discrimination biases. We have therefore focused on the perception of these features for our current study.
To address the comment, we elaborated the respective section in the main text, explaining this rationale in more detail:

Methods section (l. 98 ff.):
“We characterized all pulsed sounds according to a set of three interpretable and diverse features, which were determined by systematic literature research. In a previous study, these features have been shown to be prominently represented at the level of neuronal activity in the auditory cortex, effectively capturing different aspects of stimulus energy and temporal structure that allow to predict stimulus-specific perceptual biases[20].”

Results section (l. 560 ff.):

“These sound features have been shown to be prominently encoded in the mouse auditory cortex[20], conforming to previous studies that investigated the auditory representation of similar features[21-23].”

Reinforcement rules.
The rationale for the three reinforcement rules should be clearly articulated. Why were these specific task structures chosen, and how do they test the theoretical claims?

We apologize if we did not make this point clear enough. In our human choice task, we implemented three different reinforcement rules, each classifying the same set of sounds based on a different feature (either pulse number, or irregularity, or imbalance). With this, we tested the discriminability of the different sound features for a fixed set of stimuli, allowing us to estimate independent perceptual maps for the discrimination of each feature.
We adjusted the respective text sections, elaborating this explanation (l. 580 ff.):

“We next probed perceptual maps under task-based conditions, using a discrimination task with varying reinforcement rules. All participants completed three rounds of a two-alternative forced choice task, each of which assigned reinforcements based on one of the three different stimulus features (*pulses per second*, *irregularity of accents*, *pulse imbalance*), so that participants had to discriminate the same set of sounds, but according to varying rules (Figure 2a, Methods). Participants were instructed that each task had the same structure, but independent rules. This procedure allowed us to compare the perceptual discrimination of the same stimuli according to different, qualitatively distinct features, providing independent estimates of task-based perceptual maps for each task condition.”

Ethical considerations.
Water deprivation in mice is common but should be explicitly justified, with clear reporting of welfare monitoring procedures.

Our mouse experiments adhere to study protocols that been used extensively without problems in animal welfare and have been approved by the local authorities on animal experiments. To address the comment, we added a note on this aspect to the Methods section (l. 231 ff., 259 ff.):

“All animal experiments were performed in accordance with the German laboratory animal law guidelines for animal research and had been approved by the Landesuntersuchungsamt Rheinland Pfalz (Approval # 23 177-07/G 17-1-051).
[…]
The water deprivation followed established standard protocols motivating subjects to engage in the task without causing notable behavioral impairments[19,24,25]. Animal welfare during the deprivation periods was ensured through regular visual inspections and weight controls.”

Statistical and Analytical Concerns
Clustering and map structure.
Similarity matrix clustering is described descriptively. Statistical validation (e.g., hierarchical clustering metrics, silhouette scores) would strengthen claims about dominant clusters.

Thank you for this constructive suggestion. In fact, we conducted hierarchical clustering to identify the structure in the mean human similarity ratings (see Figure 1c with dendrogram on the right side of the plot). To address the comment and further quantify the clustering results, we added an evaluation using the Calinski-Harabasz index to our manuscript (see Results l. 551 ff. and Methods l. 414 ff.). This index serves as a measure to find the optimal number of clusters to partition data, and was maximal for a number of k=2 clusters. Thus, this analysis confirming our initial qualitative statement.

> *Regression modeling.*
> *Linear regression assumes additive, linear contributions of features. Perceptual spaces may exhibit non-linear interactions. Testing interaction terms or non-linear models would provide greater confidence. Multicollinearity diagnostics should also be reported.*

Thank you for this comment. In our analysis, we focus on a linear regression to estimate how strongly the different stimulus features – an interpretable description of stimulus properties – contribute to the task-naïve similarity ratings in our scaling paradigm. We intentionally, do not include interaction terms, as these would be difficult to interpret and therefore do not meet the aims of our analysis. However, we agree with the reviewer that we should mention how we determined that our data was suitable for a linear analysis approach and further mention the limitations of this approach explicitly.
To address the comment, we added information on the suitability of our data for a linear regression approach to the main text (see l. 436 ff.):

"Assumptions of linear regression for the pooled data were verified by inspection of residual normality (Q-Q plot), homoscedasticity (residuals vs. fitted), and absence of multicollinearity (variance inflation factor VIF=1.123), confirming the suitability of the linear model."

Moreover, we added a Limitations section to our discussion, summarizing the methodological constraints of our linear analysis approach (l. 951 ff.):

"We primarily use linear models to quantify correlations or regression analyses in our data, finding that these comparably simple statistical approaches suffice to reveal significant effects. Future studies including additional non-linear models may in the future be used to resolve finer differences in the structures of perceptual maps."

> *Learning curves.*
> *Sigmoid fits are appropriate, but goodness-of-fit metrics (R^2, AIC) should be provided and compared against alternative models (e.g., linear fits).*

Thank you for this suggestion. We followed the reviewer's comment and computed R^2 and AIC for our logistic fit, comparing it to an alternative linear model. We find that our logistic fit of learning curves explains a higher fraction of variance than the linear model for all task conditions. We added a sentence to the Methods section, reporting this point (l. 338 ff.):

"We verified that these sigmoid fits of learning curves explained our data better than an alternative simple linear model by assessing the fraction of explained variance (n=152 participants; mean $R^2 \pm$ SD: pulses, sigmoid 0.118±0.254 vs. linear 0.104±0.115, irregularity, sigmoid 0.140±0.174 vs. linear 0.090±0.105, imbalance, sigmoid 0.101±0.157 vs. linear 0.068±0.100), as well as Akaike's Information Criterion (mean

AIC ± SD: pulses, sigmoid -750±80 vs. linear -743±95, irregularity, sigmoid -620±56 vs. linear -611±50, imbalance, sigmoid -608±66 vs. linear -601±40)."

Correlation strength and multiple comparisons.
Many reported correlations are weak to moderate (e.g., RP = 0.238). Effect sizes should be discussed explicitly. Given the number of correlations performed, stricter correction procedures (e.g., Bonferroni) or bootstrapped confidence intervals would provide a more robust assessment. Non-significant correlations, particularly for imbalance, should not be overinterpreted.

For all analyses, where we repeatedly correlate the same measure, we applied a Bonferroni-Holm correction to correct for multiple testing (see l. 515).
We further expanded our Discussion section to explicitly mention the small correlations as a limitation of the study (l. 943 ff.):

"Despite showing statistical significance, the correlations between map structures across task conditions and the correlation to learning abilities in the discrimination tasks often showed only intermediate or weak effect sizes, highlighting that a substantial degree of variance in perceptual map structures might be affected by e.g. finite sampling of pairwise similarities and additional biological factors, not considered in our study."

In addition, we revised our main text, removing all interpretations of non-significant statistical results.

Feature-specific conservation.
The high cross-species correlation for pulses (RP = 0.896) is compelling. However, irregularity (RP = 0.520) and imbalance (RP = 0.222) show markedly weaker alignment. Thus, claims of "shared key structures" should be framed as feature-dependent rather than general.

Thank you for this comment. We believe there might be a misunderstanding. For our cross-species comparisons, we compare the mouse data exclusively to the human data obtained from the discrimination task where pulses per second were reinforced. Therefore, we restrict our comparisons on data from structurally equivalent tasks, and do not compare mouse data to human task in other conditions.
Nevertheless, we agree with the reviewer that we should mention explicitly in the discussion of our data that our cross-species comparisons were restricted to only one task condition and one stimulus feature. We have expanded our Limitations section accordingly (l. 938 ff.):

"In addition, compared to our human sample undergoing perceptual discrimination tasks for three different stimulus features, our mouse cohort was substantially smaller and only underwent perceptual discrimination of one single stimulus feature. Replication of our experiments, using a larger mouse sample and more extensive discrimination tasks of more stimulus features could in the future corroborate the initial observation in our cross-species comparison."

Species comparisons.
Formal statistical tests comparing human and mouse similarity matrices (e.g., Mantel tests or matrix correlations with permutation tests) would strengthen conservation claims.

For our analyses, we focus on comparing perceptual map structures through correlating the flattened off-diagonal elements of the corresponding similarity matrices. This approach follows previous studies and has been shown to efficiently capture the global structure of a representational map[20,26,27]. Please note that any observed correlation in this analysis is not trivial, but rather indicates that pairs of stimuli (each represented in one off-diagonal field of the similarity matrix) are similarly perceived, despite varying conditions in which this perception has been assessed.

> *Interpretation and Potential Circularity*
> *The finding that pulse rate dominates naïve maps and also yields fastest learning may partly reflect circularity. If the naïve perceptual space is already strongly organized around pulse rate, superior discrimination along this dimension is expected. The manuscript should discuss this possibility and clarify whether the data truly demonstrate predictive power beyond baseline feature salience.*

Thank you for bringing up this thought. Indeed, our study identified that pulses per second was dominantly reflected on task-naïve perceptual maps as well as during the discrimination task. It is however not trivial that a feature which is salient in naïve perception outside a task will also dominate task performance. An opposing hypothesis might be that the brain operates on distinct and largely unrelated maps for task-naïve and task-based conditions. It is indeed a theoretical possibility that a different feature than pulses per second would have been better represented and faster learned to discriminate as soon it would have been reinforced. Our finding is therefore not trivial.
The fact that pulses per second feature is indeed rapidly learned speaks against this opposing hypothesis and indicates a significant similarity of task-naïve and task-based perceptual maps.
We thank the reviewer for the comment and adjusted parts of our Discussion section to explain this aspect in more detail (l. 865 ff.):

"These observations are not trivial and raise the question, how perceptual maps could support decision making in a task context. Two extreme scenarios may provide a reference: (i) A general representational map has formed before the task[28] and behavior during the task depends on an optimized read-out procedure, leading to adjusted inferences from this otherwise stable representational map[1,27,29]. (ii) Learning during the task leads to the formation of a novel, separate representational map with an optimal configuration for the specific demands of the task[30-32]. These hypothetical extreme scenarios highlight that a correlation between task-based and task-naïve perception is not guaranteed. Given the psychophysical approach in our study without simultaneous recordings of neural activity, our dataset does not allow to entirely disentangle these two scenarios. Nevertheless, our findings that naïve perceptual maps show a high degree of correlation to task-based perceptual maps and task performance are consistent with the first scenario, assuming a context-dependent readout of a pre-existing representational map."

> *The relationship between "perceptual maps" and "perceptual learning" is also insufficiently clarified. Are maps pre-existing frameworks that constrain learning, or are they plastic and shaped by experience? The manuscript would benefit from situating findings within established theories of perceptual learning.*

According to our framework, perceptual learning constitutes an associative process that operates on a perceptual map and links represented items (i.e. stimuli) to specific behavioral responses to optimize classification or discrimination in order to obtain rewards. In line with this, our analyses, modeling the decision behavior of mice and humans over the course of learning, suggest that individuals dynamically

adjust their choice strategies, focusing on stimulus-related features and disregarding stimulus-independent strategies.
In the context of representational maps, as we hypothesize above, learning could involve two qualitatively distinct processes: (i) an optimization of the representational structures per se to allow a quick and improved readout of the map, and (ii) an optimization of readout strategies to couple particular sensory representations with a given behavior, with our observation primarily supporting the latter.
We thank the reviewer for the constructive comment. To address it, we expanded our Discussion section to elaborate on this aspect (l. 928 ff.):

"A flexible re-mapping of behavioral responses to the different features represented on the same perceptual map could disentangle reinforced features from non-reinforced features according to the current demands of an individual, allowing for behavioral adaptation despite maintained value- and rule-related codes in higher cortical areas[33-35]. To allow this adaptation, perceptual learning likely includes on the one side, an optimization of the representational structures of a map to allow a quick readout, and on the other side, an optimization of readout strategies to couple sensory representations with different behaviors."

Cross-Species and Personality Analyses
The link between perceptual similarity weights and personality measures is intriguing but under-theorized. What is the proposed neurobiological mechanism linking similarity structure to creativity or personality traits? Moreover, if conservation across species is emphasized, how would analogous personality constructs be operationalized in rodents?

The reviewer raises an interesting question, which is nevertheless not at the prime focus of the manuscript presented here. Here, we primarily compared average perceptual structures in humans and mice.
In a previous study in humans, we had investigated the correlation between individual representational structures and personality traits. There, we have shown that perceived similarities between semantic stimuli, but also non-semantic auditory stimuli can predict individual creative performance[26]. This finding aligns with previous studies[36,37], suggesting that individuals that perceive different stimuli as more similar have improved abilities to form associations between these stimuli and thus develop creative thoughts[19]. In animals, this enhanced associative ability might manifest as an ability to learn new tasks more quickly, or adapt more quickly to novel environmental demands. Future studies will be needed to address this aspect in detail.
To address the comment, we expanded the respective section of our discussion to address this point (l. 885 ff.):

"We did not observe a significant interaction of task-based map structures and basic personality traits. However, it was recently shown that humans who perceive a higher total degree of similarity between semantic or auditory stimuli also tend to think more creatively[26,36,38]. This observation suggests that perceptual similarity between different stimuli might be an important factor of their associability, so that individuals that form stronger perceptual links are more likely to develop innovative solutions. In future translational studies, detailed characterizations of perceptual maps might allow to investigate creativity mechanistically in the context of learning behavior, providing a potential bridge between human and non-human research[19]."

Species differences in GLM parameters (e.g., initial biases in mice) should be discussed explicitly. Are these methodological (head-fixed vs. free-moving) or biological?

Here, we are wondering why the reviewer suggests to discuss head fixation as a relevant parameter that should be discussed explicitly, as neither the human participants nor the mice involved in our study were undergoing head fixation at any time of the experiment.

Nevertheless, the reviewer is right in pointing out that the behavioral assays in humans and mice are designed to be similar, but in the end of course not identical. It therefore cannot be excluded that methodological differences may play into the different behavioral components captured by the parameters of the regression model. To address the comment, we adjusted parts of our Discussion section (l. 920 ff.):

"Our analysis revealed that initial mouse behavior was dominated by stimulus-independent choice factors. In contrast, initial human behavior was dominated by stimulus-related factors right from the beginning that, however, needed to be re-adjusted during learning to match the current reinforcement rule. Although the psychophysical approaches in humans and mice used in this study were largely comparable in structure, some differences in the task design were unavoidable. This implies that apart from the neurobiological differences between the species, causing mice to take much longer than humans to adjust their perceptual decisions and learn the discrimination, some methodological differences could also contribute."

> *Discussion and Limitations*
> *The Discussion primarily reiterates results rather than synthesizing broader implications. It would benefit from engagement with:*
> *• perceptual learning theory,*
> *• representational geometry approaches,*
> *• and potential neural correlates (e.g., future neuroimaging or calcium imaging studies).*
>
> *Limitations should be more fully acknowledged, including:*
> *• student-biased human sample,*
> *• potential order effects,*
> *• limited mouse sample size,*
> *• and restriction of cross-species comparison to one acoustic feature.*

We appreciate the reviewer's suggestion. Following this comment and previous comments, we substantially expanded our Discussion section to cover the mentioned aspects (see p. 28-30). Moreover, we added a Limitations section to the discussion, commenting on the points raised by the reviewer (l. 935 ff.):

"Limitations:
Various limitations need to be considered for the interpretation of our study. Our human sample was limited to student participants and showed an uneven gender distribution, potentially reducing the generalizability of our findings to other populations. In addition, compared to our human sample undergoing perceptual discrimination tasks for three different stimulus features, our mouse cohort was substantially smaller and only underwent perceptual discrimination of one single stimulus feature. Replication of our experiments, using a larger mouse sample and more extensive discrimination tasks of more stimulus features could in the future corroborate the initial observation in our cross-species comparison. Despite showing statistical significance, the correlations between map structures across task conditions and the correlation to learning abilities in the discrimination tasks often showed only intermediate or weak effect sizes, highlighting that a substantial degree of variance in perceptual map

structures might be affected by e.g. finite sampling of pairwise similarities and additional biological factors, not considered in our study. Furthermore, our study design of assessing task-based perceptual maps under different conditions for the same stimuli does not allow to disentangle if the correlations between map structures fully result from shared representations, or from structural relations between the stimuli in our library. Even though, we did not find strong correlations between sound features in our stimulus set, future generalization studies with independent stimulus sets will be necessary to solve this issue. We primarily use linear models to quantify correlations or regression analyses in our data, finding that these comparably simple statistical approaches suffice to reveal significant effects. Future studies including additional non-linear models may in the future be used to resolve finer differences in the structures of perceptual maps."

Reviewer #2:

This paper studied the perceptual similarity to a set of pulsed sound stimuli using psychophysics measurement under both naive and task contexts, and compared the perception between humans and mice, showing interesting cross-species conservation. The study provides a valuable demonstration and dataset of investigating perceptual changes during learning, computing comparable perceptual map across different behavioral paradigms, and linking different species for their shared cognitive processes.

Thank you.

1. Line 76: Is this perceptual similarity rating stable? What is the reliability of the rating? Measuring perceptual similarity with a scale is more subject to fluctuations and drifts of individual criterion and such, when compared to paradigms like match-to-sample.

Thank you for raising this question. In our study, each participant iteratively rates the similarity between all pairs of the 20 sounds in our stimulus library, corresponding to 210 trials. Thus, we only obtain one rating of each stimulus pair per participant, limiting our analyses to cross-sectional approaches without the possibility to investigate retest-reliability or systematic fluctuations over time. In a previous study, however, we have demonstrated that the representational structure assessed from simple perceptual ratings is robust against normalizations to individual biases and dynamic ranges[26].
In addition, please note that in order to avoid confounding due to changing task engagement over time, we randomize the sequence of presented stimuli for each individual. Thus, the overall structure of perceptual maps in individuals should be comparable and average perceptual structures are expected to be unbiased by sequence effects.
To address the reviewer's comment, we added an additional note to the main text to make this point clearer (l. 546 f.):

"Note that the order of stimuli was randomized to avoid confounding of the perceptual ratings by sequence biases."

2. Line 99-102: The lack of pairwise correlations between stimulus features suggests that the stimulus is approximately orthogonal, but does not imply independent perceptual encoding. If the goal is to assess the feature independency in perceptual encoding, explicit implementing interaction terms in the regression model is needed, which would affect the interpretation of other results.

We appreciate this constructive criticism. We agree with the reviewer that the lack of a significant correlation between stimulus features does not allow to infer independent representations. In fact, we have recently studied the neuronal representation of pulsed sound stimuli in the mouse auditory cortex, observing an entanglement of the representations of the three stimulus features[20]. We have therefore adjusted our wording in the manuscript accordingly to correct this statement (l. 116 f.):

"We correlated the feature values of our full set of sounds (mouse study) and the subset of sounds (human study), respectively, observing only weak associations between the different sound features.
[…]
In a previous study, we used *in vivo* two-photon calcium imaging of population activity patterns in the auditory cortex of passively listening mice in response to the same set of pulsed sound stimuli, and found that the representational structure at the neuronal level captures entangled properties of the pulsed sounds described by the three stimulus features and reflects major aspects of the perceptual map[20]."

The main goal of our regression analysis was to estimate the contribution that each individual stimulus feature has on the structure of task-naïve perceptual maps. We intentionally did not include regression terms, as these would be difficult to interpret in the context of our analysis aim.
Nevertheless, we agree with the reviewer that we should include additional information on the suitability of our data for linear regression, including measures of normality, homoscedasticity, and multicollinearity. We have added a statement on these aspects to our manuscript (l. 436 ff.) (see also response to a comment from reviewer 1 on p. 8 of rebuttal letter):

"Assumptions of linear regression for the pooled data were verified by inspection of residual normality (Q-Q plot), homoscedasticity (residuals vs. fitted), and absence of multicollinearity (variance inflation factor VIF=1.123), confirming the suitability of the linear model."

> *3. Line 151: In future studies, it will be interesting to collect verbal description of rules more frequently to see when the correct rule emerges and how it corresponds to the performance improvement.*

This is indeed a very interesting idea. We added a note on this aspect to the Discussion section of our manuscript (l. 858 ff.):

„Learning abilities were correlated with explicit insight in the rules of the task, but a notable fraction of individuals also performed well in the task without explicit understanding. In the future, it will be interesting to assess task insight in greater detail, combining longitudinal assessments of understanding over the course of learning with specific instructions to facilitate or hinder this insight."

> *4. Line 199: Were the initial learning trials under tasks excluded when calculating the difference in response probabilities? Same question for mouse data. What's the criterion if any?*

For the human data, we computed the mean response probabilities averaging over all available trials (n=170) in each task. For the mouse data, we computed the mean response probabilities, considering all trials after mice had learned the initial discrimination (i.e. showing a performance ≥70% for two consecutive sessions, see Methods l. 294 f.). The reason for these differences lies in the fact that human learning in the

task was much faster compared to mice, and often reached a plateau performance quickly, so that we believe that the quantification for each species is reasonable.
Motivated by the reviewer's comment, we revised parts of our Results and Methods sections to make this point clearer (l. 402 ff.):

"Specifically, we computed the mean response probabilities for each sound in the task. For the human data, we used the data of each choice task to build the mean over all trials in which a given stimulus was presented. For the mouse data, we did the same, however using only the data after the mice had learned the initial discrimination, showing an average performance ≥70% for two consecutive sessions."

Human Results part (l. 684 ff.):
"For each pair of sounds, we computed the difference in mean response probabilities over all trials in the task, and used the complement of this metric as a measure of task-based perceptual similarity."

Mouse Results part (l. 738 f.):
"In analogy to our human analyses, we constructed task-based similarity matrices from the average poking probabilities in the auditory go/no-go task after mice had successfully learned the initial task."

> *5. The reported similarity of perceptual maps across contexts (naive vs task) and across species is interesting. However, with the current experiment, it is difficult to disentangle whether the observed similarity is driven by the underlying structure of the stimulus features, or by the internal perceptual maps. The map similarity/predictability is mainly quantified with correlation, while a high correlation score here might be a reflection of the shared stimulus features, or be dominated by one of the features (pulse number). It would be helpful to more explicitly address the contribution of stimulus features in the analysis, as well as if they have tested generalization, such as by predicting the response to an independent stimulus set.*

Thank you for raising these questions. We agree that it is difficult with our experimental setup to disentangle whether perceived similarities are driven by independent perceptual representations for the different stimulus features, or by overlaps between stimulus features. In the end, likely both may be true, given our observation of prevalent, but weak correlations between stimulus features within our selected stimulus library. Indeed, it would be highly interesting to test how generalizable naïve perceptual weights for specific stimulus features are in predicting choice behavior for independent stimuli. However, such an experiment would not allow to directly compare the structure of task-naïve and task-based perceptual maps, as we did it in our current set, using the exact same stimuli in both conditions.
To address the reviewer's comment, we added this point as a limitation to our Discussion section (l. 947 ff.):

"Furthermore, our study design of assessing task-based perceptual maps under different conditions for the same stimuli does not allow to disentangle if the correlations between map structures fully result from shared representations, or from structural relations between the stimuli in our library. Even though, we did not find strong correlations between sound features in our stimulus set, future generalization studies with independent stimulus sets will be necessary to solve this issue."

6. Line 358: Could the authors please elaborate on why the results support speed-accuracy tradeoffs (SAT)? SAT is a decision strategy, either innate or task-induced, where speed and accuracy are inversely related. Here I don't observe such a tradeoff.

We apologize if our line of reasoning as briefly touched upon in the discussion is unclear. We find that the representation of the different stimulus features in task-naïve perceptual maps varies and that those features with a stronger representation are easier to discriminate in a choice task.
According to the framework of speed-accuracy tradeoffs, task performance may be affected by both, the sensory certainty (i.e. the representation) about the type of a particular sensory input, as well as the time that an individual takes to decide for the type of input. Since the response times in our choice task were constrained, we assume that accuracy in the task should mostly depend on the degree to which a feature is represented.

However, we agree with the reviewer that this aspect is rather indirect and involves several assumptions and we therefore decided to exclude this from the discussion.

7. Line 369-372: To me, the findings in this paper seem to be equally consistent with both scenarios. The correlation between naive and task-based maps could also arise from the constraints on how easily perceptual maps can be transformed during learning. The boundary between 'representational map formation' and 'readout process' is difficult to draw based on behavioral data alone, as the two are relative concepts that are functionally and even neurally intertwined. Depending on the hierarchical level of the stimulus representation, the timescale of 'remodeling' may be fast or slow, making the claim in line 375-377 less compelling as evidence against representational change. It's good that the authors made it explicit that due to the lack of neural data, they cannot fully distinguish the two scenarios mentioned in line 363-367.

We are happy to hear that the this point of the discussion is appreciated. Picking up on the reviewer's comments, we added a note on the mentioned possibility of quickly re-structuring maps (l. 882 ff.):

"Consistent with the employment of a pre-existing map, we observed a fast and flexible adaptation of perceptual choice behavior to varying task contingencies in humans within comparably few trials. Theoretically, this observation could also result from rapidly restructured representational maps, however, in the light of the general tendency of stability in basic perceptual representations, such a strong remodeling appears unlikely[39]."

8. The participants were told there were 3 task rules but NOT told what the 3 rules were, correct? Could mention it early on in the method.

Yes, exactly. The participants were instructed that the three tasks had independent rules. We followed the suggestion made by the reviewer and added a note on this aspect to the Methods and Results section (l. 188 f., 584 f.).

9. I understand this paper focused on learning, but it would be interesting in future studies to test humans with explicit instructions on what the task rules are and make comparisons. Some category-learning processes, such as medical image learning or chord learning, usually require prolonged

training and explicit guidance. This situation might be more comparable to the pulse imbalance condition, and could be quantitatively or even qualitatively different from learning to discriminate features that are already highly discernable from naive perceptual space.

This is a very interesting thought, and we agree that it would be interesting to address this point in future studies. We added a note on this outlook to our Discussion section (l. 859 f.).

"In the future, it will be interesting to assess task insight in greater detail, combining longitudinal assessments of understanding over the course of learning with specific instructions to facilitate or hinder this insight."

10. Line 694-701: In the quantification of the explicit understanding, have the authors considered/controlled in anyway for the difficulty of task rule description? I would imagine describing the pulse number rule is quite easy while the other two is tricky to articulate. Especially the example reports for the irregularity of accents and pulse imbalance appear vague and potentially have conceptual/expressive overlap across rules. This poses challenges in interpreting the results in line 157

We assessed task understanding through free text responses that participants submitted after each round of the discrimination task to explain the respective task rule. For the evaluation of this data, we simplified the verbal responses into binary variables, representing whether the respective task was understood or not. The evaluation of the free text responses was performed in a blinded fashion by a fixed expert of the study team, and was repeated two times to ensure reliability of the evaluation.
Still, we agree with the reviewer that our binarization simplified the actual understanding of individuals, and that other more detailed assessments might have resulted in more nuanced descriptions of task insight. However, the aspect of explicit versus implicit task understanding was also not at the center of our study, which rather focused on assessments of perceptual map structures under different conditions.
We nevertheless thank the reviewer for bringing up this point, and we added more detailed information on the evaluation of the free text responses to the Methods section (l. 381 f.).

"The evaluation of the free text responses was performed in a blinded fashion by an expert of the study, and was repeated two times to ensure reliability of the evaluation."

In addition, we added a link to the manuscript allowing to access the public repository containing the study data and code: https://doi.org/10.12751/g-node.bgb999.

We believe that the adjustments in response to the reviewers' comments improve the quality and clarity of our manuscript.
Thank you for taking the time to review.

The authors

References:

1 Chambers, A. R., Aschauer, D. F., Eppler, J.-B., Kaschube, M. & Rumpel, S. A stable sensory map emerges from a dynamic equilibrium of neurons with unstable tuning properties. *Cerebral Cortex* **33**, 5597-5612, doi:10.1093/cercor/bhac445 (2023).

2 Pancholi, R., Ryan, L. & Peron, S. Learning in a sensory cortical microstimulation task is associated with elevated representational stability. *Nature Communications* **14**, 3860, doi:10.1038/s41467-023-39542-x (2023).

3 Aschauer, D. F. *et al.* Learning-induced biases in the ongoing dynamics of sensory representations predict stimulus generalization. *Cell Reports* **38**, 110340, doi:10.1016/j.celrep.2022.110340 (2022).

4 Liberti, W. A., Schmid, T. A., Forli, A., Snyder, M. & Yartsev, M. M. A stable hippocampal code in freely flying bats. *Nature* **604**, 98-103, doi:10.1038/s41586-022-04560-0 (2022).

5 Drieu, C. *et al.* Rapid emergence of latent knowledge in the sensory cortex drives learning. *Nature* **641**, 960-970, doi:10.1038/s41586-025-08730-8 (2025).

6 Shepard, R. N. & Chipman, S. Second-order isomorphism of internal representations: Shapes of states. *Cognitive Psychology* **1**, 1-17, doi:10.1016/0010-0285(70)90002-2 (1970).

7 Mead, A. Review of the Development of Multidimensional Scaling Methods. *Journal of the Royal Statistical Society. Series D (The Statistician)* **41**, 27-39, doi:10.2307/2348634 (1992).

8 Burns, B. & Shepp, B. E. Dimensional interactions and the structure of psychological space: The representation of hue, saturation, and brightness. *Perception & Psychophysics* **43**, 494-507, doi:10.3758/BF03207885 (1988).

9 Kriegeskorte, N. & Wei, X.-X. Neural tuning and representational geometry. *Nature Reviews Neuroscience* **22**, 703-718, doi:10.1038/s41583-021-00502-3 (2021).

10 Noda, T., Aschauer, D. F., Chambers, A. R., Seiler, J. P. H. & Rumpel, S. Representational maps in the brain: concepts, approaches, and applications. *Frontiers in Cellular Neuroscience* **18** (2024).

11 Mur, M. *et al.* Human Object-Similarity Judgments Reflect and Transcend the Primate-IT Object Representation. *Front Psychol* **4** (2013).

12 King, M. L., Groen, I. I. A., Steel, A., Kravitz, D. J. & Baker, C. I. Similarity judgments and cortical visual responses reflect different properties of object and scene categories in naturalistic images. *NeuroImage* **197**, 368-382, doi:10.1016/j.neuroimage.2019.04.079 (2019).

13 Huth, Alexander G., Nishimoto, S., Vu, An T. & Gallant, Jack L. A Continuous Semantic Space Describes the Representation of Thousands of Object and Action Categories across the Human Brain. *Neuron* **76**, 1210-1224, doi:10.1016/j.neuron.2012.10.014 (2012).

14 Kriegeskorte, N. *et al.* Matching Categorical Object Representations in Inferior Temporal Cortex of Man and Monkey. *Neuron* **60**, 1126-1141, doi:10.1016/j.neuron.2008.10.043 (2008).

15 Hanks, T. D. & Summerfield, C. Perceptual Decision Making in Rodents, Monkeys, and Humans. *Neuron* **93**, 15-31, doi:10.1016/j.neuron.2016.12.003 (2017).

16 Manley, G. A. Comparative Auditory Neuroscience: Understanding the Evolution and Function of Ears. *Journal of the Association for Research in Otolaryngology* **18**, 1-24, doi:10.1007/s10162-016-0579-3 (2017).

17 Fritzsch, B. *et al.* Evolution and development of the tetrapod auditory system: an organ of Corti-centric perspective. *Evolution & Development* **15**, 63-79, doi: 10.1111/ede.12015 (2013).

18 Lipovsek, M. & Elgoyhen, A. B. The evolutionary tuning of hearing. *Trends in Neurosciences* **46**, 110-123, doi: 10.1016/j.tins.2022.12.002 (2023).

19 Seiler, J. P. H. *et al.* Towards Circuit Mechanisms of the Creative Process. *Creativity Research Journal*, 1-20, doi:10.1080/10400419.2026.2646676 (2026).

20 Seiler, J. P. H., Cazzetta, G., Noda, T. & Rumpel, S. Representational geometry of sounds in the auditory cortex explains biases in perceptual decision-making in mice. *Cell Reports* **45**, doi:10.1016/j.celrep.2026.117221 (2026).

21 Fox, N. P., Leonard, M., Sjerps, M. J. & Chang, E. F. Transformation of a temporal speech cue to a spatial neural code in human auditory cortex. *eLife* **9**, e53051, doi:10.7554/eLife.53051 (2020).

22 Weber, A. I. *et al.* Spatial and temporal codes mediate the tactile perception of natural textures. *Proceedings of the National Academy of Sciences* **110**, 17107-17112, doi:10.1073/pnas.1305509110 (2013).

23 Bagur, S. *et al.* A spatial code for temporal information is necessary for efficient sensory learning. *Science Advances* **11**, eadr6214, doi:10.1126/sciadv.adr6214 (2025).

24 Bathellier, B., Ushakova, L. & Rumpel, S. Discrete Neocortical Dynamics Predict Behavioral Categorization of Sounds. *Neuron* **76**, 435-449, doi:10.1016/j.neuron.2012.07.008 (2012).

25 Bathellier, B., Tee, S. P., Hrovat, C. & Rumpel, S. A multiplicative reinforcement learning model capturing learning dynamics and interindividual variability in mice. *Proceedings of the National Academy of Sciences* **110**, 19950-19955, doi:10.1073/pnas.1312125110 (2013).

26 Seiler, J. P. H., Elpelt, J., Ghobadi, A., Kaschube, M. & Rumpel, S. Perceptual and semantic maps in individual humans share structural features that predict creative abilities. *Communications Psychology* **3**, 30, doi:10.1038/s44271-025-00214-9 (2025).

27 Noda, T. *et al.* Homeostasis of a representational map in the neocortex. *Nature Neuroscience*, doi:10.1038/s41593-025-01982-7 (2025).

28 Eppler, J.-B., Kaschube, M. & Rumpel, S. Statistical learning and representational drift: A dynamic substrate for memories. *Current Opinion in Neurobiology* **94**, 103107, doi:10.1016/j.conb.2025.103107 (2025).

29 Stevens, J.-L. R., Law, J. S., Antolík, J. & Bednar, J. A. Mechanisms for Stable, Robust, and Adaptive Development of Orientation Maps in the Primary Visual Cortex. *The Journal of Neuroscience* **33**, 15747, doi:10.1523/JNEUROSCI.1037-13.2013 (2013).

30 Polley, D. B., Steinberg, E. E. & Merzenich, M. M. Perceptual Learning Directs Auditory Cortical Map Reorganization through Top-Down Influences. *The Journal of Neuroscience* **26**, 4970, doi:10.1523/JNEUROSCI.3771-05.2006 (2006).

31 Jehee, J. F. M., Ling, S., Swisher, J. D., van Bergen, R. S. & Tong, F. Perceptual Learning Selectively Refines Orientation Representations in Early Visual Cortex. *The Journal of Neuroscience* **32**, 16747, doi:10.1523/JNEUROSCI.6112-11.2012 (2012).

32 Caras, M. L. & Sanes, D. H. Top-down modulation of sensory cortex gates perceptual learning. *Proceedings of the National Academy of Sciences* **114**, 9972-9977, doi:10.1073/pnas.1712305114 (2017).

33 Reinert, S., Hübener, M., Bonhoeffer, T. & Goltstein, P. M. Mouse prefrontal cortex represents learned rules for categorization. *Nature* **593**, 411-417, doi:10.1038/s41586-021-03452-z (2021).

34 Miller, K. J., Botvinick, M. M. & Brody, C. D. Value representations in the rodent orbitofrontal cortex drive learning, not choice. *eLife* **11**, e64575, doi:10.7554/eLife.64575 (2022).

35 Zhang, M. *et al.* The representation of decision variables in orbitofrontal cortex is longitudinally stable. *Cell Reports* **43**, doi:10.1016/j.celrep.2024.114772 (2024).

36 Benedek, M. *et al.* How semantic memory structure and intelligence contribute to creative thought: a network science approach. *Thinking & Reasoning* **23**, 158-183, doi:10.1080/13546783.2016.1278034 (2017).

37 Kenett, Y. N. & Faust, M. A Semantic Network Cartography of the Creative Mind. *Trends in Cognitive Sciences* **23**, 271-274, doi:10.1016/j.tics.2019.01.007 (2019).

38 Kenett, Y. N., Anaki, D. & Faust, M. Investigating the structure of semantic networks in low and high creative persons. *Front Hum Neurosci* **8** (2014).

39 Xin, Y. *et al.* Sensory-to-Category Transformation via Dynamic Reorganization of Ensemble Structures in Mouse Auditory Cortex. *Neuron* **103**, 909-921.e906, doi:10.1016/j.neuron.2019.06.004 (2019).

Rebuttal Letter: Manuscript "Auditory perceptual maps in humans and mice share common structures and predict perceptual decisions in discrimination learning" – Review 2

With this, we would like to re-submit our manuscript entitled "Auditory perceptual maps in humans and mice share common structures and predict perceptual decisions in discrimination learning" (ID COMMSPSYCHOL-26-0085-T) for publication as a research article in *Communications Psychology*.

We thank the reviewers for their positive feedback.
The minor revisions to address the comments of reviewer 1 are marked by blue font color in the attached main text and in this rebuttal letter.

In the following, we address the comments point by point:

> ***Reviewer #1***
> *Minor revisions. The manuscript is substantially improved. The authors addressed the conceptual ambiguity around "perceptual map," added proper statistical validation of the clustering, compared sigmoid fits to linear alternatives, and expanded the Discussion to engage with perceptual learning theory.*
> *Two small issues remain: the self-report hearing screening should be acknowledged as a limitation of the psychophysical validity of the data, and the asymmetry in trial exclusion across species should be noted explicitly. Neither requires new data or reanalysis. Both can be resolved with one or two sentences in the Limitations section. Congratulations on your nice paper!*

Thank you. We adjusted the limitations section of the manuscript to elaborate on the mentioned aspects (l. 936 ff.):

"Various limitations need to be considered for the interpretation of our study. Our human sample was limited to student participants and showed an uneven gender distribution, potentially reducing the generalizability of our findings to other populations. Hearing abilities were tested via self-reports only. In addition, compared to our human sample undergoing perceptual discrimination tasks for three different stimulus features, our mouse cohort was substantially smaller and only underwent perceptual discrimination of one single stimulus feature. The tasks in humans were shorter than the mouse experiment, leading to an asymmetry in the number of trials considered in our analyses."

> ***Reviewer #2***
> *I have no further comments. The authors have addressed my concerns by including more information, adjusting the wording, and explicitly incorporating some of the limitations of their study.*

Thank you.

We appreciate taking the time to review.

The authors
